# Sign of the Gap Temperature Dependence in CsPb(Br,Cl)_3_ Nanocrystals Determined by Cs-Rattler-Mediated Electron–Phonon Coupling

**DOI:** 10.1021/acs.jpclett.4c03491

**Published:** 2025-01-23

**Authors:** Shima Fasahat, Nadesh Fiuza-Maneiro, Benedikt Schäfer, Kai Xu, Sergio Gómez-Graña, M. Isabel Alonso, Lakshminarayana Polavarapu, Alejandro R. Goñi

**Affiliations:** † Institut de Ciència de Materials de Barcelona, ICMAB-CSIC, Campus UAB, 08193 Bellaterra, Spain; ‡ CINBIO, Universidade de Vigo, Materials Chemistry and Physics Group, Dept. of Physical Chemistry, Campus Universitario Lagoas Marcosende, 36310 Vigo, Spain; ¶ ICREA, Passeig Lluís Companys 23, 08010 Barcelona, Spain

## Abstract

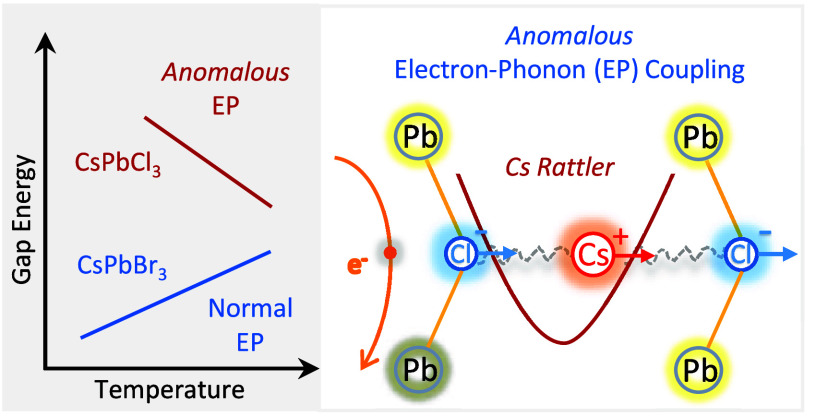

So far, the striking sign reversal in the near-ambient slope of the gap temperature dependence of colloidal CsPbCl_3_ perovskite nanocrystals (NCs) compared to its Br counterpart remains unresolved. Pure bromide NCs exhibit a linear gap increase with increasing temperature, to which thermal expansion and electron–phonon interaction equally contribute. In contrast, the temperature slope for the chlorine compound gap is clearly negative. By combining temperature- and pressure-dependent photoluminescence on a series of CsPb(Br_1–*x*_Cl_*x*_)_3_ NCs, we unravel the origin of such inversion. The electron–phonon interaction is solely responsible, undergoing a sudden change in sign and magnitude due to activation of an anomalous electron–phonon coupling mechanism linked to vibrational modes characterized by synchronous octahedral tilting and *Cs rattling*. This takes place in the shrunken orthorhombic NC lattice for Cl concentrations exceeding ca. 40%. We have thus clarified a puzzling result directly impacting the optoelectronic properties of lead halide perovskite NCs.

Colloidal nanocrystals (NCs) of lead halide perovskites of the type APbX_3_, with organic or inorganic A-site cation and halide substitution on the X site, exhibit very high photoluminescence (PL) quantum yields, high color purity, large band gap tunability, while being produced by low-cost, solution-processed methods.^1−3^ In this respect, fundamental knowledge about the band gap of the active material and its temperature dependence becomes mandatory for the optimization of optoelectronic devices. Under ambient conditions, metal halide perovskites, whether in bulk or nanocrystalline form, show a fairly linear gap increase with increasing temperature for either tetragonal or cubic phases. This gap temperature dependence is almost ubiquitous in halide perovskites but restricting ourselves to NCs, it can be found in MAPbI_3_,^4,5^ FAPbI_3_,^6,7^ MAPbBr_3_,^8−11^ CsPbI_3_^12−14^ and CsPbBr_3_.^12,13,15−18^ A notable exception is CsPbCl_3_, which exhibits a sign reversal in the temperature slope, i.e. the gap decreases with increasing temperature near ambient, both for NCs^12^ and thin films.^19^

Recently, another hydrid perovskite with a similar temperature dependence of negative gap slope has been found, namely MHyPbBr_3_, where MHy stands for methylhydrazinium.^20^ This material was purposely synthesized as part of a series of lead bromides obtained by varying the A-site cation, so as to achieve a Goldschmidt tolerance factor^21^*t* smaller, equal and larger than one for CsPbBr_3_, MA_0.13_EA_0.87_PbBr_3_ and MHyPbBr_3_, respectively. Here, MA stands for methylammonium and EA for ethylammonium, while the exact composition was adjusted to obtain *t* ≈ 1. The underlying idea^20^ is that the stereochemical expression of the lead 6*s*^2^ lone pair, measured by the magnitude of the Pb off-center displacement, is predetermined by the tolerance factor. From their X-ray diffraction results and electron localization function calculations they conclude that CsPbBr_3_ (*t* < 1) is totally stereo inactive, MA_0.13_EA_0.87_PbBr_3_ (*t* = 1) is only dynamically stereo active and solely MHyPbBr_3_ (*t* > 1) exhibits static stereo activity. The main hypothesis is that the sign and magnitude of the electron–phonon interaction, as reflected by the slope of the gap temperature dependence, is given by the strength of the lone pair expression. Their own data support this hypothesis, for the gap temperature slope of CsPbBr_3_ is small but positive, that of MA_0.13_EA_0.87_PbBr_3_ is also positive but large and, strikingly, MHyPbBr_3_ exhibits a clear negative slope. Regrettably, given that CsPbCl_3_ has almost the same tolerance factor than its bromide counterpart, i.e. it should be stereo inactive, the negative temperature slope of its gap cannot be explained that way. Moreover, there are several lead-free perovskites showing positive slopes,^22−24^ despite being much more prone to exhibit static lone pair expression.

The effects of a temperature variation on the band structure of any semiconductor are described by the thermal expansion (TE) and electron–phonon (EP) interaction terms.^25−27^ The former arises from the intrinsic anharmonicity of the crystal potential, which typically leads to expansion or contraction of the crystal lattice when temperature is raised or lowered, respectively. The gap thus partly changes due to the temperature-induced volume variations. The renormalization of the electronic states due to lattice vibrations is essentially produced by smearing of the crystal potential and by scattering of electrons by phonons. Both effects are proportional to the Bose–Einstein phonon occupation number and so is the EP term.^28,29^ Although shown for MAPbI_3_,^30^ most lead halide perovskites also display a positive temperature slope of the gap which is due to similarly strong thermal expansion and electron–phonon interaction effects. Near ambient, the gap pressure coefficient of perovskites is negative, as determined by the bonding/antibonding and atomic orbital character of the band extrema.^31^ Thus, the TE contribution to the total temperature slope is positive. It has been shown^30^ that the EP term is usually described by a single Einstein oscillator to account for the main peak in the phonon density of states. Since its amplitude is positive, the EP contribution to the temperature renormalization of the gap is also positive. However, a recent report indicates that the incorporation of low amounts of Cs into the MAPbI_3_ lattice leads to an *anomalous* electron–phonon coupling, that induces a reduction of the gap temperature slope.^32^ The only way to account for such anomalous coupling is to introduce an additional Einstein oscillator with *negative* coupling constant. Here we are thus set to investigate if a similar but stronger effect could be at the origin of the negative temperature slope of the gap in CsPbCl_3_ NCs.

In this Letter, we show results of combined temperature and pressure-dependent PL measurements, performed on a series of colloidal CsPb(Br_1–*x*_Cl_*x*_)_3_ mixed-anion NCs to unravel the origin of the aforementioned reversal in the slope of the gap temperature dependence. The NC composition was varied continuously by ionic exchange in the primal colloidal solution. Careful analysis of the PL data allowed us to disentangle thermal expansion and electron–phonon interaction effects on the gap temperature dependence. We found that, concomitant with a transition into an orthorhombic phase, occurring for Cl contents around 40%, the electron–phonon interaction undergoes a sudden and radical change in sign and magnitude. In contrast, thermal expansion effects remain the same. Based on recent observations in mixed-cation Cs_*x*_MA_1–*x*_PbI_3_ single crystals,^32^ we interpret such behavior as due to the activation of an anomalous electron–phonon coupling mechanism involving Cs-cation degrees of freedom, which takes place in the shrunken orthorhombic phase stable at high Cl concentrations. The vibrational modes leading to the anomalous coupling are the so-called Cs rattlers, which have been recently invoked to understand the origin of the extremely low thermal conductivity of CsPbBr_3_.^33^

First, pure CsPbBr_3_ perovskite NCs were synthesized through ligand-assisted tip-ultrasonication, as described elsewhere^34,35^ (see also the Supporting Information (S.I.) for details). The prepared NCs were nearly monodisperse nanocubes with an average size ranging from 8 to 10 nm (see Figure S1 of the S.I.). Mixed-halide CsPb(Cl,Br)_3_ NCs were obtained by a ligand exchange strategy,^36^ where the composition was varied continuously by performing the ionic exchange in the primal colloidal solution, until the desired emission wavelength was achieved. Figure S2 of the S.I. displays the variation of the emission color under UV illumination, resulting from the strong dependence of the NC electronic states on halide composition. In fact, the Cl content of the NCs after ionic exchange was estimated from the energy of the PL peak maximum by linear interpolation between the values for pure Br^35^ and pure Cl^12^ perovskite NCs, as described in Note #1 of the S.I.

The structural characterization of the mixed-halide NCs has been carried out at room temperature by X-ray diffraction. Figure 1 shows the two-theta scans of NC samples with different compositions but similar to the ones studied here. The assignment of the two main peaks has been performed according to the experimental and theoretical grazing-incidence wide-angle X-ray scattering (GIWAXS) results of bulk CsPbBr_3_.^37^ The sudden shift of the diffraction peaks to higher 2θ values, occurring for Cl concentrations between 30% and 40% (see Figure 1) is a clear indication of a structural phase transition into a phase with smaller unit cell volume. A comparison with GIWAXS^37^ indicates that the sharp, nonsplit (100) and (200) diffraction peaks for low Cl contents are characteristic of the *cubic* (α) phase. In contrast, at high Cl concentrations the shrunken phase exhibits a broader (200) peak but a relatively sharp (100) peak with two satellites. The broadening of the (200) peak is likely due to a not well-resolved splitting (see Figure S3 in Note #1 of the S.I.). Both changes in the X-ray diffraction line shape are indicative of a transition into the *orthorhombic* (γ) phase.^37^ The existence of the cubic phase for low Cl contents is somewhat unexpected, for at ambient conditions the stable phase of bulk CsPbBr_3_ is the orthorhombic γ phase.^37,38^ However, high-resolution TEM studies of individual CsPbBr_3_ NCs unraveled a size-dependent transition temperature.^39^ For NCs with sizes below approximately 10 nm, strain relaxation can favor the stabilization of the cubic α phase at room temperature, when the Cs dynamics is unfolded.^37,40^ In fact, our Raman results obtained at ambient for the whole series of mixed-halide NCs (see Figure S4 of the S.I. and ref (41)) give strong support to the interpretation of the X-ray data.

**Figure 1:**
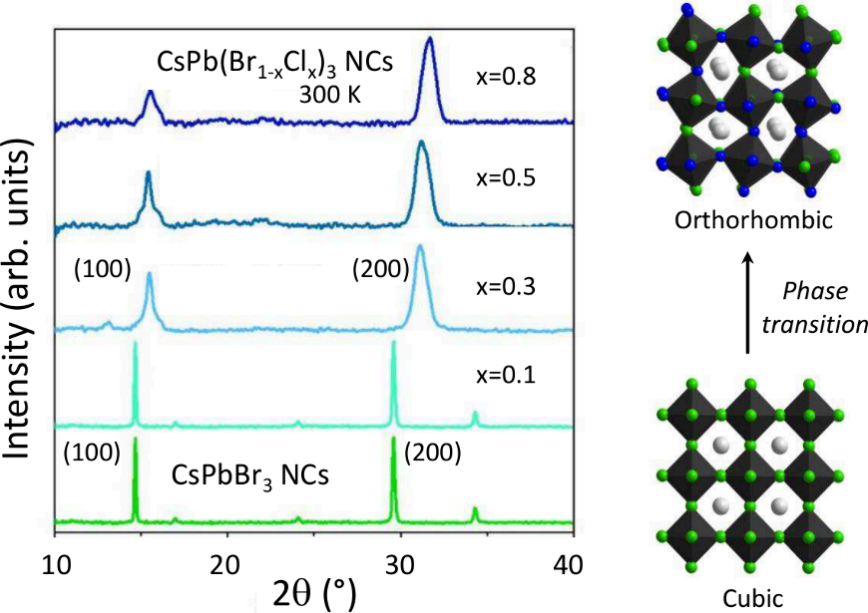
Room temperature X-ray diffraction patterns (λ = 1.5406 Å) of NC samples with similar compositions as the ones studied here. Crystal structures are also displayed (Br, Cl and Cs atoms in green, blue and gray, respectively).

The temperature dependent PL measurements were carried out in vacuum using a gas-flow cryostat,^30^ whereas the high-pressure PL measurements were performed at room temperature employing a gasketed diamond anvil cell (DAC) with anhydrous propanol as pressure transmitting medium.^31^Figures 2a and 2b display the temperature evolution of the PL spectra of the CsPb(Br_1–*x*_Cl_*x*_)_3_ NCs with *x* = 0.25 and 0.75, respectively, from 300 to 80 K. All spectra were normalized to its absolute maximum intensity and vertically offset for clarity. The values of the PL peak maximum obtained from a line-shape analysis of the PL spectra fits are plotted as a function of temperature in Figure 2c.^30,31^ For practical purposes we consider the shift of the PL peak energy with temperature (or pressure) to be representative of the shift of the gap.^5,42^ The red lines in Figure 2c represent the result of a linear fit to the data points around room temperature (results for other compositions are shown in Figures S5a-c and 6a-c of the S.I.). The slopes of all gap-vs-temperature curves are plotted as a function of halide composition in Figure 3a and listed in the first column of Table 1. The most striking result of this work concerns the sudden change in sign of the temperature slope, from positive, like in MAPbI_3_,^30^ to negative, like for CsPbCl_3_ NCs,^12^ for Cl concentrations either lower or higher than 40%, respectively. We notice that the sign turnover seems to coincide with the occurrence of the structural phase transition from cubic to orthorhombic with increasing Cl content (see Figure 1).

**Figure 2:**
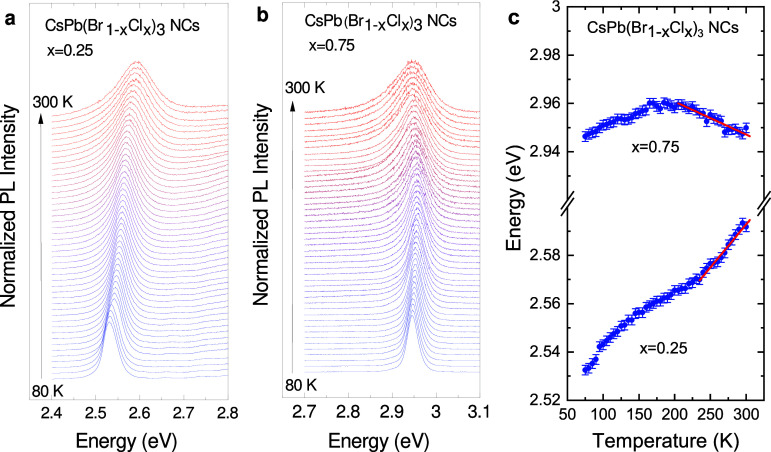
PL spectra of CsPb(Br_1–*x*_Cl_*x*_)_3_ NCs with (a) *x* = 0.25 and (b) *x* = 0.75 measured at different temperatures between 80 and 300 K in steps of 5 K, using the 405 and 355 nm laser line for excitation, respectively. The spectra were normalized to their maximum intensity and shifted vertically for clarity. (c) Plot of the temperature dependence of the maximum peak position obtained from the PL spectra of (a) and (b) (blue symbols). The red lines correspond to linear fits to the data points around room temperature.

**Figure 3:**
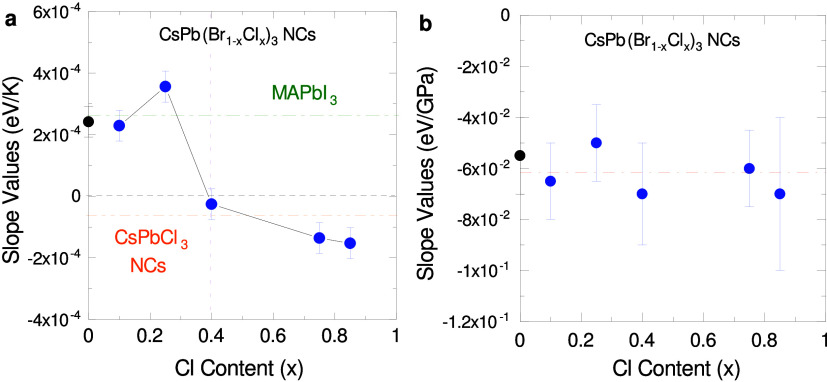
Slope values of the linear function of the gap energy versus (a) temperature and (b) pressure near ambient conditions of the CsPb(Br_1–*x*_Cl_*x*_)_3_ NCs. The values for bulk MAPbI_3_^30^ and CsPbCl_3_ NCs^12^ are indicated with dot-dashed lines. Black symbols are from ref (35). The red dot-dashed horizontal line in (b) corresponds to the average of the pressure slopes.

**Table 1:** Measured Linear Gap Temperature  and Pressure  Coefficients for *x* = 0, 0.1, 0.25, 0.4, 0.75, and 0.85^a^

*x*	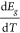 (10^–4^ eV/K)	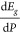 (eV/GPa)	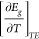 (10^–4^ eV/K)	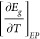 (10^–4^ eV/K)	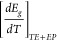 (10^–4^ eV/K
0*	2.3(5)	–0.055(15)	1.3(4)	0.9(2)	2.2(3)
0.10	2.2(5)	–0.065(15)	1.5(5)	0.8(2)	2.3(3)
0.25	3.1(2)	–0.050(15)	1.2(5)	1.8(2)	3.0(3)
0.40	0.3(5)	–0.070(20)	1.9(4)	1.0	0.3(3)
				–2.6(2)	
0.75	–1.4(5)	–0.060(15)	1.6(4)	1.0	–1.4(3)
				–4.0(2)	
0.85	–1.5(5)	–0.070(30)	1.9(7)	1.0	–1.2(3)
				–4.1(2)	

a Also listed are the values of the TE term , computed after eq 1, and the results from least-squares fits to the first derivative data points corresponding to the EP terms  and the total temperature renormalization . Numbers in parentheses are error bars. The asterisk indicates data are from ref (35).

To understand such a behavior we have first to disentangle the effects of thermal expansion and electron–phonon interaction on the gap temperature renormalization.^5,30,32^ According to eq 1 in Note #2 of the S.I., the derivative of the gap over temperature contains solely the thermal expansion (TE) term and the one due to electron–phonon interaction (EP).^25,26,43^ The effect on the gap due to the lattice contraction with decreasing temperature is intimately related to the response of the electronic states under hydrostatic pressure:^25,26^
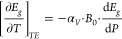
1where −α_*V*_ is the volumetric thermal expansion coefficient, *B*_0_ is the bulk modulus and  is the pressure coefficient of the gap, determined here from high pressure experiments.

The gap pressure coefficient was determined at room temperature from PL spectra recorded at different pressures, as displayed for all Cl contents in Figure S7a–e in Note #4 of the S.I. Figure S8 shows the variation with pressure of the gap energy, as obtained from PL line-shape fits, in the stability range of the ambient pressure phase. The slopes of the linear fits to the data points (red solid lines in Figure S8) are plotted as a function of Cl content in Figure 3b and listed in Table 1. Surprisingly, for all NC compositions the gap pressure coefficient  is approximately the same in sign and magnitude, with an average value of (−60 ± 15) meV/GPa, represented by the red dashed line in Figure 3b (for a complete survey of the gap pressure and temperature coefficients of lead halide perovskites see ref (35)). Hence, with increasing temperature thermal expansion always causes a gradual opening of the gap, independent of chlorine content. To calculate the TE term according to eq 1, we used the values of the volumetric thermal expansion coefficient α_*V*_ = (1.14 ± 0.05) × 10^–4^K^–1^ and (1.26 ± 0.05) × 10^–4^K^–1^ from bulk CsPbBr_3_ and CsPbCl_3_ for low and high Cl contents, respectively,^44^ and the same bulk modulus of *B*_0_ = (21 ± 5) GPa from CsPbBr_3_^45^ for all compositions. Table 1 contains the so-computed TE term values. Consequently with the steadiness of the gap pressure coefficients, the TE contributions are quite independent of the halide composition, being all positive and similar in magnitude, within experimental uncertainty. This implies that the sign reversal comes from changes in the electron–phonon interaction.

Regarding the contributions to the gap temperature renormalization stemming from electron–phonon interactions, the most important ones arise from peaks in the phonon density of states (DOS).^26^ This is at the origin of the Einstein-oscillator model,^43,46,47^ which approximates the different contributions to the EP term by effective oscillators with effective amplitude *A*_*i*_ and phonon eigen-frequency ω_*i*_, inferred from the peaks in the phonon DOS (see eq (6) in Note *⧧*5 of the S.I.). The EP correction to the gap is obtained by calculating analytically the first derivative of the Bose–Einstein occupation number with respect to temperature as
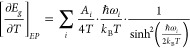
2Here *n*_*B*_(ω_*i*_,*T*) = (*e*^βℏω_*i*_^ – 1)^−1^ with  stands for the Bose–Einstein phonon occupation factor of the *i*th oscillator.

To evaluate the EP term we thus have to calculate numerically the first derivative of the gap energies over temperature on a point-by-point basis. To avoid unwanted amplification of the scatter of the first-derivative data points, we slightly smoothed the data sets prior to derivation using a five-points average method (see Figure S9a–e of Note #5 of the S.I.). The dark-green symbols in Figure 4a,b correspond to the first derivative data sets for a Cl content of *x* = 0.25 and 0.75; two representative compositions below and above the turnover composition, respectively (see Figure S10a–c of the S.I. for the resting compositions). To obtain the EP term (or terms) by fitting, *only* the data points represented by closed symbols in Figures 4a,b and Figure S10a–c were considered. These are the data points within the range of linearity, coinciding with the temperature range of the red lines in Figure 2c and Figure S9a–e (see Note #3 of the S.I. on the importance of linearity). The blue dot in Figure 4a,b corresponds to the TE term resulting from eq 1 and tabulated in Table 1. The blue dot-dashed line indicates that the TE term is temperature independent, at least in the linearity range. The contribution from electron–phonon interaction is calculated using the function of eq 2. As for the archetypal perovskite MAPbI_3_,^30^ for Cl contents lower than the turnover composition at ca. 40% a single Einstein oscillator is sufficient to account for the EP term. For MAPbI_3_ the oscillator has a positive amplitude and a frequency of 6 meV.^30^ An inspection of the phonon DOS for the three methylammonium lead halide (Cl, Br, and I) compounds^48^ indicates that the oscillator frequency of 6 meV lies slightly above the first well-defined peak or band in the DOS, corresponding to acoustical and low-frequency optical modes (see Note #6). Since these are all phonons of the inorganic cage, we can safely assume that a similar oscillator will account for the EP term in the mixed-halide NCs as well. We have thus fixed the oscillator frequency, leaving only its amplitude as adjustable parameter. The function of eq 2 together with the constant contribution from TE were fitted to the data points (only closed symbols) of Figures 4a,b and Figures S10a-c. The resulting amplitudes are listed in Table S2 (S.I.). The solid black curves and the dashed red curves represent the resulting total rate of gap renormalization per Kelvin and the EP contributions to it, respectively.

**Figure 4:**
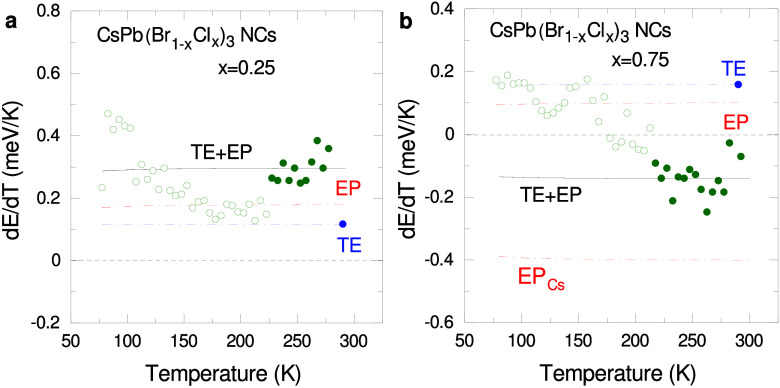
First derivative of the gap energy with respect to temperature (dark-green symbols), numerically calculated from the smoothed data of Figure 2c for CsPb(Br_1–*x*_Cl_*x*_)_3_ NCs with (a) *x* = 0.25 and (b) *x* = 0.75. The solid black lines represent a fit to *only* the closed data points, using the sum of thermal expansion (TE, blue dot-dashed curve and symbol) and electron–phonon interaction (EP, red dot-dashed curve). In (b), the curve labeled EP_*Cs*_ represents the additional Einstein oscillator introduced to account for the anomalous EP coupling. See text for details.

For Cl contents higher than the turnover concentration the situation with the EP term changes dramatically. Due to the fact that the TE term and the EP contribution from the coupling to the inorganic cage phonons are both positive, the only way to attain a negative temperature derivative is to introduce an additional Einstein oscillator with negative amplitude. We thus used for the EP term two Einstein oscillators: One with both fixed frequency and (positive) amplitude as for the single oscillator case, and one with adjustable (negative) amplitude but frequency fixed to a value of 4 meV, the frequency of the Cs rattle modes^33^ (see discussion below). The red dot-dashed curves labeled EP_*Cs*_ in Figure 4b and Figure S10b,c represent the contribution of the additional Einstein oscillator to the EP term.

The results of the least-squares fits are shown in Table S2 of Note #5 in the S.I. Strikingly, the amplitude of the additional oscillators with fixed (4 meV) rattler frequency ranges from −10 to −20 meV, thus overcompensating thermal expansion and the normal EP term. We recall that the most intense Raman modes have frequencies below 100 cm^–1^ (ca. 12.5 meV),^48,49,50^ thus, a frequency of 4 meV matches well the vibrational spectrum of the inorganic cage. Finally, in Table 1 the values computed at room temperature of the TE term, the two contributions to the EP term and the total temperature renormalization of the gap (TE+ *∑*_*i*_EP_*i*_) are listed for all studied compositions. As a consistency check, we note the excellent agreement between the experimental temperature slopes of the gap and the values obtained from the fits to the first derivative data points (first and last column of Table 1, respectively).

About the possible origin of the anomalous EP coupling term, we provide an explanation supported by similar observations made by the incorporation of small amounts of Cs in two Cs_*x*_MA_1–*x*_PbI_3_ single crystals (*x* = 0.05 and 0.1).^32^ In the Cs-containing samples and above a certain onset temperature of around 260 K, the slope of the linear temperature dependence of the gap reduces to about half the value of MAPbI_3_. Such behavior was explained by the introduction of an extra Einstein oscillator with negative amplitude associated with the appearance of an additional electron–phonon coupling mechanism. The latter was attributed to dynamic tilting of the PbI_6_ octahedrons in *synchrony* with the translational dynamics of the Cs cations between equivalent potential minima of the cage voids; a dynamics unfolded above the onset temperature. For the CsPb(Br,Cl)_3_ NCs we will propose a similar interpretation but with nuances, accounting for differences in crystal structure and halide ionic radius.

Recently, the constant-energy surfaces of the atomic potential energy were calculated by density functional theory as a function of octahedral tilting angle for CsPbI_3_ and CsSnI_3_.^22,51,52^ In the orthorhombic phase, as for the NCs with high Cl contents, these potential energy surfaces exhibit a multiwell landscape.^22,51^ The key point is that the shrunken lattice of the orthorhombic phase leaves very little space for the Cs cations to move inside the cage voids. Thus, even though they are locked, the Cs cations oscillate around the minimum of the atomic potential. These oscillations are the aforementioned *Cs rattlers*, to which the extremely low thermal conductivity of CsPbBr_3_ have been attributed.^33^ The molecular dynamics (MD) calculations of the lattice thermal conductivity show that its strong reduction arises from lattice anharmonicities resulting from the effect of dynamic disorder introduced by Cs rattlers. In their analysis, the Cs cations are interpreted as damped Einstein oscillators with an effective frequency of 4 meV.^33^ Furthermore, phonon dispersion calculations of a system with artificially raised Cs masses demonstrate an increased interference of the Cs rattling with the acoustic phonon modes. Our interpretation, now supported by the MD calculations, is that the close proximity to the halide anions makes the Cs rattlers to become coherently coupled to matching vibrational modes of the inorganic cage involving octahedral tilting. The origin of the anomalous EP coupling are these coupled modes, which provide additional oscillator strength to the phonon DOS, represented by the additional Einstein oscillator. In contrast, at low Cl concentrations, where NCs crystallize in the cubic phase, the mean tilt angle is zero and the unfolded Cs dynamics is limited to the central region of the cage voids. This, together with the larger cage size, could be the cause of the loss of coherence, leading to the suppression of the anomalous EP coupling. We refer to Note *⧧*7 of S.I. for clarification, where we also provide a tentative explanation for the negative sign of the anomalous EP term based on the Fröhlich interaction.^53^

In conclusion, we elucidated the reason for the sign reversal of the temperature slope of the gap, when comparing CsPbCl_3_ NCs (negative slope) with their bromide counterparts (positive slope). For this purpose a series of CsPb(Br_1–*x*_Cl_*x*_)_3_ NCs with five different Cl contents were synthesized by anionic exchange. Using temperature and pressure-dependent PL, we univocally disentangle the contributions from thermal expansion and electron–phonon interaction. The aforementioned slope sign turnover is triggered by the occurrence of a structural phase transition, taking place at a Cl concentration of ca. 40%, and the effects this transition has on the nature of the EP coupling term. For low Cl concentrations the NCs are cubic^39^ and exhibit around room temperature a linear gap temperature dependence with the typical positive slope of MAPbI_3_.^30^ Here the EP term accounts for the ”normal” coupling to the inorganic cage phonons. On the contrary, for orthorhombic NCs with high Cl concentrations the gap temperature slope is negative as for CsPbCl_3_ NCs.^12^ The only way to account for the sign reversal is to introduce an additional EP term with negative amplitude, which becomes the leading term in the gap renormalization. We ascribe it to an *anomalous* coupling to mixed modes arising from Cs rattler modes intermixed with cage vibrations involving octahedral tilting. Given the relevance of the gap temperature dependence for the optoelectronic properties of perovskite NCs, its correct assessment is key for the advancement of emergent photovoltaics and efficient light emission and/or sensing devices, for instance.

## Data Availability

All data generated or analyzed during this study are either included in this published article and its Supporting Information files or are available from the corresponding author on reasonable request.

## Supporting Information Available

Details about the employed materials and the synthesis of the NCs, including the way in which the variation of the halide composition was performed by ionic exchange, details of the PL and Raman characterization of the entire series of CsPb(Br,Cl)_3_ NCs, in particular the determination of the NC composition from the energy of the PL peak maximum, cascade plots of the PL spectra and the gap-energy data points as a function of temperature and pressure for the samples with the Cl concentrations not shown in the manuscript, the smoothed energy-vs-temperature data point sets for all compositions and the first-derivative plots for the three Cl contents not shown in the main text, table with the parameters resulting from the least-squares fits to the first derivative data points, and a detailed discussion of the underlying physics of the normal and anomalous EP term (PDF)
Transparent Peer Review report available (PDF)
