# Supplementary material for: Sign of the Gap Temperature Dependence in CsPb(Br,Cl)3 Nanocrystals Determined by Cs-Rattler-Mediated Electron–Phonon Coupling
Source: J Phys Chem Lett. 2025 Jan 23;16(4):1134–41. doi: 10.1021/acs.jpclett.4c03491 (PMC11915369; doi:10.1021/acs.jpclett.4c03491)
Supplement: Supplementary file 1 — jz4c03491_si_001.pdf [file jz4c03491_si_001.pdf]

Supporting Information

Sign of the Gap Temperature Dependence in $CsPb(Br,Cl)_3$ Nanocrystals Determined by Cs-Rattler-Mediated Electron-Phonon Coupling

Shima Fasahat,† Nadesh Fiuza-Maneiro,‡ Benedikt Schäfer,† Kai Xu,† Sergio Gómez-Graña,‡ M. Isabel Alonso,† Lakshminarayana Polavarapu,‡ and Alejandro R. Goñi*,†,¶

†*Institut de Ciència de Materials de Barcelona, ICMAB-CSIC, Campus UAB, 08193 Bellaterra, Spain*

‡*CINBIO, Universidade de Vigo, Materials Chemistry and Physics Group, Dept. of Physical Chemistry, Campus Universitario Lagoas Marcosende, 36310 Vigo, Spain*

¶*ICREA, Passeig Lluís Companys 23, 08010 Barcelona, Spain*

E-mail: goni@icmab.es

Note ♯0: Synthesis of the colloidal nanocrystals (NCs)

Materials

Cesium carbonate (Cs_2CO_3, 99.9%), lead (II) bromide ($PbBr_2$, >98%), lead (II) chloride ($PbCl_2$, 99%), 1-octadecene ($C_{18}H_{36}$, 90%), oleic acid ($C_{18}H_{34}O_2$, 90%), and oleylamine ($C_{18}H_{37}N$, 70%) were purchased from Merck. Hexane solutions were purchased from Millipore-Sigma. All chemicals were used as received.

Preparation of precursor solutions

The cesium oleate (CsOL) precursor solution was prepared by dissolving 407.0 mg of cesium (I) carbonate (1.25 mmol) in 20 mL of octadecene, along with 1.25 mL of oleic acid, under vigorous stirring at 130 °C. The reaction between the carbonate and the acid produces a white precipitate, which dissolves only at temperatures exceeding 120 °C. Therefore, the precursor must be heated prior to use. For the preparation of the $PbCl_2$ solution 1 mmol of $PbCl_2$, 1.5 mL of oleylamine, 1.5 mL of oleic acid, and 100 mL of hexane were loaded in a 250 mL Pyrex bottle. The mixture was heated to 60 °C under vigorous stirring until the $PbCl_2$ completely dissolved. The resulting $PbCl_2$ solution in hexane was used as the chloride source for synthesizing mixed-halide perovskite nanocrystals (NCs).

$CsPbBr_3$ perovskite NCs synthesis

In a typical synthesis,[1] 15 mL of octadecene, 1.5 mL of oleic acid, 1.5 mL of oleylamine, and the precursor powders (1 mmol of Cs_2CO_3 and 3 mmol of $PbBr_2$) were placed in a 50 mL glass vial. The reaction mixture was then subjected to tip ultrasonication (SONOPULS HD 3100, BANDELIN) at a power of 30 W for 30 minutes. During the process, a visible color change from

colorless to yellow indicated the formation of perovskite NCs. The resulting solution was purified through centrifugation at 10,000 rpm for 10 minutes, and the pellet was redispersed in 6 mL of hexane. A second centrifugation step was performed at 5,000 rpm for 10 minutes to remove larger particles. The supernatant was collected and diluted further for the preparation of mixed-halide perovskite NCs.

Tuning NC halide stoichiometry

Mixed halide $CsPb(Br_{1-x}Cl_x)_3$ NCs were obtained via a halide-exchange reaction using a $PbCl_2$ solution. In a typical procedure, 500 μL of the previously synthesized $CsPbBr_3$ NC colloidal dispersion was transferred to a 25 mL glass vial and diluted with 1.5 mL of hexane. The $PbCl_2$ solution was then added incrementally under vigorous stirring until the photoluminescence (PL) emission shifted to the desired wavelength. The progress of the reaction was monitored by PL, with the observed blue shift in PL emission confirming the halide exchange process.

Note ♯1: Characterization of the colloidal NCs: Transmission electron microscopy (TEM), X-ray diffraction (XRD), photoluminescence (PL) & Raman

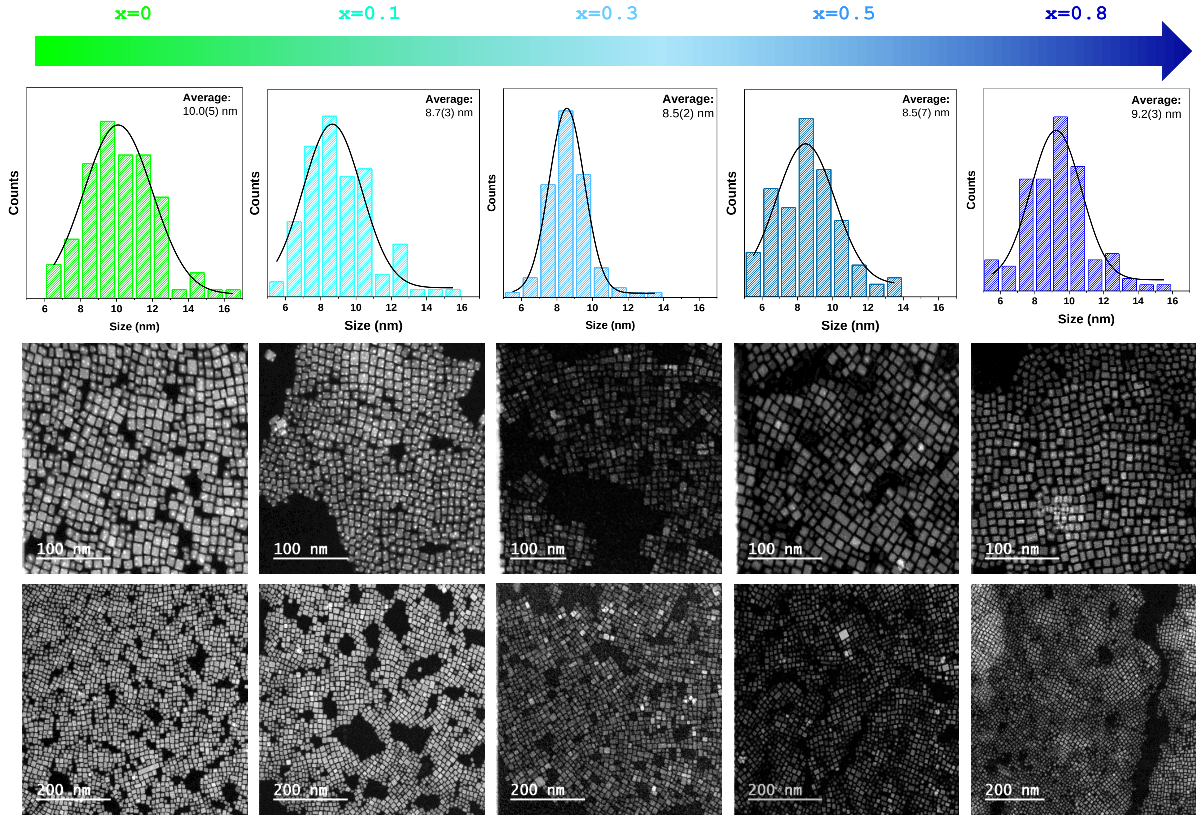

Figure S 1: Scanning TEM micrographs of the $CsPb(Br_{1-x}Cl_x)_3$ NCs with the indicated concentrations (same samples as the ones of X-ray diffraction shown in Fig. 1 of the manuscript). Histograms displaying the NC size distribution by sampling ca. two hundred NCs. Lateral size values correspond to average of both rectangle sides. Numbers in parentheses correspond to the standard deviation of the indicated average value of each NC ensemble with more than hundred NCs.

In Fig. S1, we show scanning TEM images and the corresponding size distribution histograms for the samples with the indicated compositions, i.e. the same as for the X-ray diffraction measurements. By sampling more than two hundred NCs in each case, we obtained a mean size of the NCs observed in low magnification TEM micrographs of Fig. S1, computed as the average of the two tetragon-side lengths (numbers in parentheses are error bars). The ionic exchange procedure typically leads to a slight decrease in NC size, induced by the partial substitution of the bromide by chloride.

The $CsPb(Br_{1-x}Cl_x)_3$ nanocrystals (NCs) exhibit bright light emission, whose wavelength strongly depends on halide composition, as displayed in Fig. S2 for ultraviolet (UV) illumination. In fact, the energy of the PL peak maximum at ambient temperature and pressure conditions can be used to estimate the average Cl concentration of the NCs by linear interpolation between the emission energies for pure Br (x=0)[2] and pure Cl (x=1)[3] NCs. The corresponding energy values and the inferred Cl contents are tabulated in Table S1.

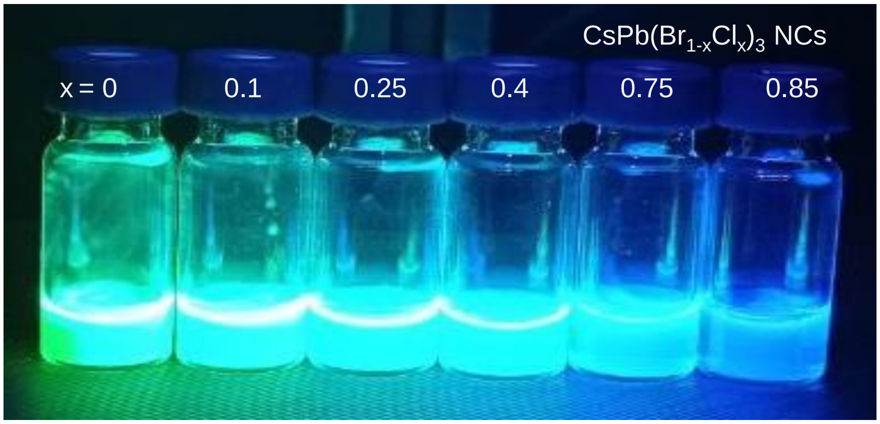

Figure S 2: Glass jars containing the studied colloidal suspensions of $CsPb(Br_{1-x}Cl_x)_3$ NCs with the indicated concentrations. Under UV illumination, the variation of the emission color with Cl composition is clearly appreciated.

The PL spectra were excited, depending on Cl concentration, with either the violet 405 nm or

Table S 1: Chlorine content (x) from the energy of the PL peak maximum at ambient conditions calculated by linear interpolation between the value of 2.435(5) eV for pure Br (x=0)[2] and 3.100(5) eV for pure Cl (x=1)[3] $CsPb(Br1-xCl_x)_3$ NCs. Number in parentheses represent error bars.

Sample	PL_{max} (eV)	x
♯1	2.505(5)	0.10(5)
♯2	2.590(5)	0.25(5)
♯3	2.710(5)	0.40(5)
♯4	2.950(5)	0.75(5)
♯5	3.000(5)	0.85(5)

the ultraviolet 355 nm line of two different solid-state lasers using a very low incident light power of ca. 1 μW (a power density below 15 W/cm^2) to avoid photo-degradation of the samples. Spectra were collected using a 20× long working distance objective with NA=0.35 and dispersed with a high-resolution LabRam HR800 grating spectrometer equipped with a charge-coupled device detector. PL spectra were corrected for the spectral response of the spectrometer by normalizing each spectrum using the detector and the 600-grooves/mm grating characteristics.

The line-shape analysis of the XRD patterns shown in Fig. 1 of the manuscript was performed by least-squares fitting using Gaussian-Lorentzian cross-product functions. Two representative examples are displayed in Fig. S3; one of the cubic phase for the pure bromide sample ($x = 0$) and the other for a Cl concentration of $x = 0.3$, for which the NCs possess an orthorhombic crystal structure. For the cubic phase both the (100) and (200) peaks are described by a single peak with a half width of ca. 0.1 deg. (red curves in Fig. S3). Due to the lifting of the three-fold degeneracy when the symmetry of the perovskite unit cell is reduced from cubic to orthorhombic, both diffraction peaks split into three components each with a half width of 0.2 deg. (grey curves in Fig. S3; red curves are the sum of the three components). Hence, at high Cl concentrations the shrunken phase exhibits a much broader (200) peak but a relatively sharp (100) peak with two satellites. The large width of the (200) peak is only apparent and is due to the unresolved splitting

of the three non-degenerate components. There is a broadening of the diffraction peaks when transforming into the orthorhombic phase, but it is much less pronounced, as might be inferred by considering the (200) peak as a single one.

At this point, we would like to comment on the absence of strong diffraction at 2θ = 21 deg. (e.g. PDF card ICSD 29073). Typically, this peak is reported in the literature as the one with the highest intensity. However, numerous studies have shown that the intensity of the peak corresponding to the (110) plane can diminish significantly[4,5] and, in some instances, become nearly imperceptible.[6] This phenomenon can be attributed to the formation of superlattices[7] resulting from the drop-casting of the NCs onto the glass substrate. Consequently, the alignment of the crystals in a specific orientation may obscure certain information about their crystal faces.

Regarding the observation that for low Cl concentrations the NCs are cubic, despite the fact that bulk $CsPbBr_3$ is orthorhombic,[8] we point out that apart from size effects,[9] as mentioned in the manuscript, there might also be a dependence of the stabilized crystal structure on the method of synthesis and post-treatment. In fact, we would like to mention that $CsPbBr_3$ NCs produced by ball milling[10] or hot injection[11,12] do crystallize in the orthorhombic phase. However, recent reciprocal space X-ray total scattering measurements on $CsPbBr_3$ NCs of different average sizes down to 5 nm indicate that in the smallest size NCs more symmetric octahedral arrangements are favored as compared to bulk.[13] This finding may reconcile, at least partially, the debate on the role of small sizes in room-temperature stabilization of the cubic crystal polymorph (thermodynamically stable at high temperature).

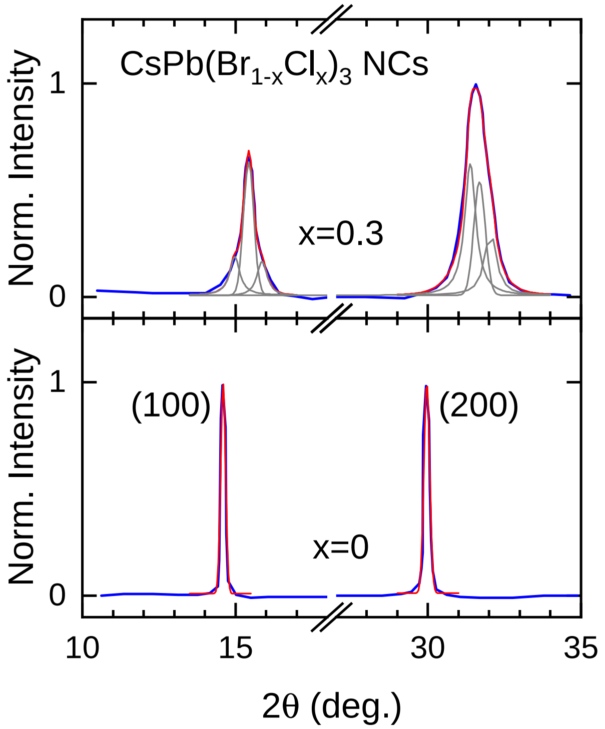

Figure S 3: Room temperature XRD patterns (blue curves) recorded with an incident wavelength of λ=1.5406 Å for $CsPb(Br_{1-x}Cl_x)_3$ NCs with the indicated concentrations. For $x = 0$ the red curves correspond to the result of least-squares fits using a single Gaussian-Lorentzian cross-product function for each peak. For $x = 0.3$ the red curves are the sum of three components (grey curves).

Figures S4(a)-(e) show the Raman spectra of NC samples with different chlorine content, as indicated, recorded at ambient temperature and pressure conditions using the 785-nm laser line for excitation. The spectral range shown corresponds to that of the inorganic cage phonons. As reported elsewhere,[14,15] their linewidth is an indicator of whether the A-site cation dynamics, Cs in the present case, is unfolded or not. This is because an unleashed A-site cation dynamics, as occurs in the cubic and tetragonal phases of metal halide perovskites, leads to strong phonon anharmonicities which, in turn, are reflected in strong broadening of the inorganic cage phonons. In addition, strong polar fluctuations induced by the A-site cation dynamics give rise to a strong Raman peak centered at zero cm^{-1}, which is usually confused with Rayleigh scattering because the

zero peak is observed as a steep increasing background towards the laser position.[16] Both effects are clearly seen in the Raman spectra of the low Cl content NC samples in Figs. S4(a)-(c), for the strongly broadened phonons are apparent as soft undulations of the steep zero Raman peak. This is strong evidence that the crystal phase of low Cl content NCs is indeed cubic or pseudo-cubic, for which the Cs cations are freely moving inside the inorganic cage voids. In frank contrast and as depicted in Figs. S4(d),(e), for the two higher Cl concentrations the zero Raman peak has disappeared and the cage phonons are better resolved as peaks, due to the strong reduction of the broadening. The latter is a direct consequence of the locking of the Cs cations inside the inorganic cage voids,[14,15] i.e. meaning that the NCs transformed into an orthorhombic phase as the Cl content increased above a value of around 30% to 40%. All this is in very good agreement with the results from X-ray diffraction.

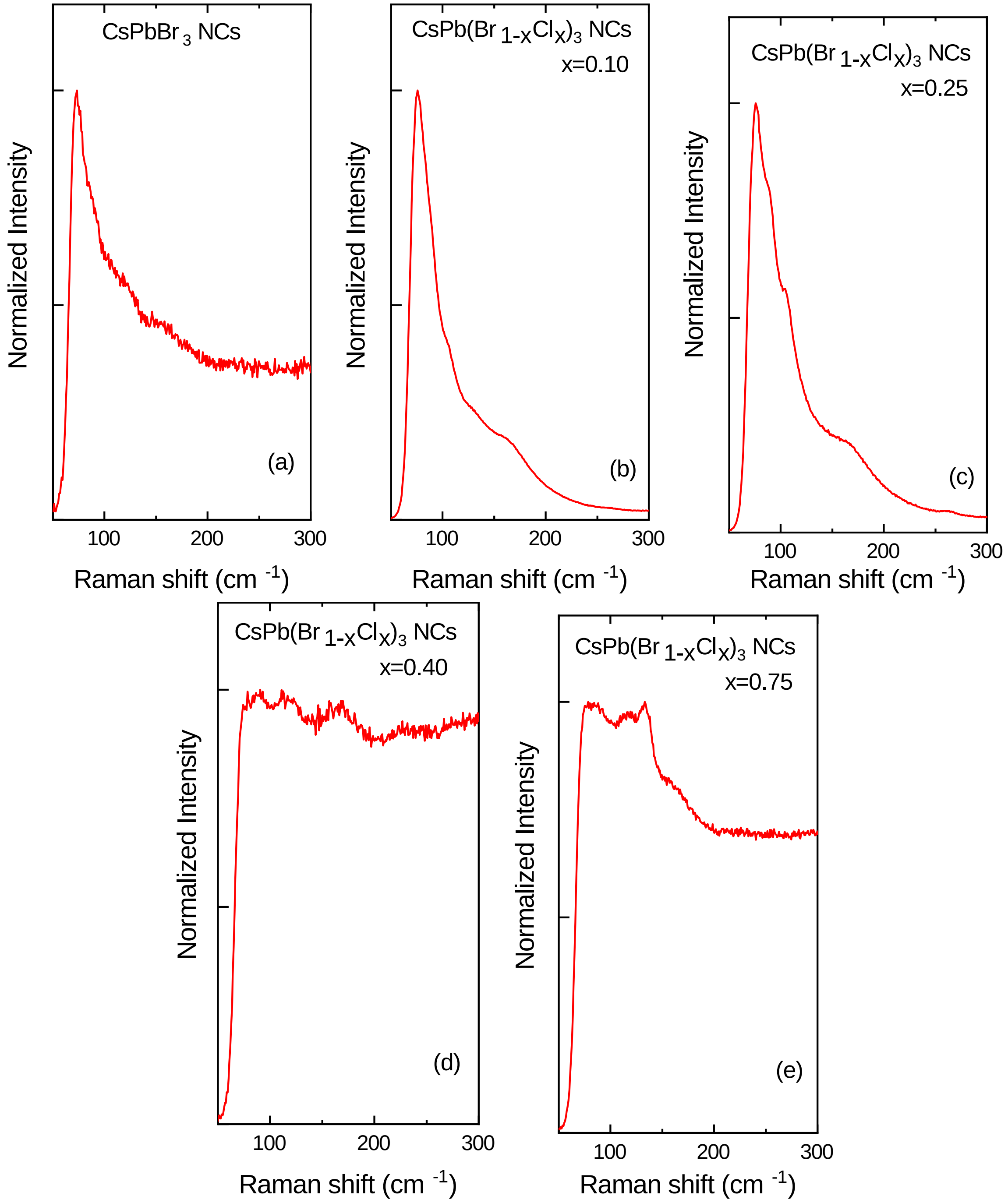

Figure S 4: Raman spectra in the spectral range of the inorganic cage phonons recorded with 785-nm excitation at ambient temperature and pressure conditions for $CsPb(Br_{1-x}Cl_x)_3$ NCs with chlorine content (a) x=0, (b) 0.10, (c) 0.25, (d) 0.40 and (e) 0.75.

Note ♯2: The temperature renormalization of the gap

The PL spectra cascade recorded for different temperatures between 80 and 300 K for the other three compositions, not shown in the main manuscript, are shown in Figs. S5a-c. The PL emission is dominated by radiative exciton recombination, which means that the maximum peak position is representative of the energy of the gap of the NCs, provided one can neglect small changes with temperature in the already small exciton binding energy of lead halide perovskites.[17] These values are plotted in Figs. S6a-c as a function of temperature for the same compositions. The red lines represent the results of linear least-squares fits to the data points around room temperature. The most striking result of this work concerns the sudden change in sign of the linear slope of the temperature renormalization of the gap, which seems to occur for Cl contents around 40%.

The derivative of the gap over temperature contains two terms; one accounts for thermal expansion effects (TE) and the other corresponds to the renormalization directly caused by electron-phonon interaction (EP), which includes the Debye-Waller and self-energy corrections:[18–20]

$$\frac{dE_g}{dT} = \left[\frac{\partial E_g}{\partial T}\right]_{TE} + \left[\frac{\partial E_g}{\partial T}\right]_{EP}. \tag{1}$$

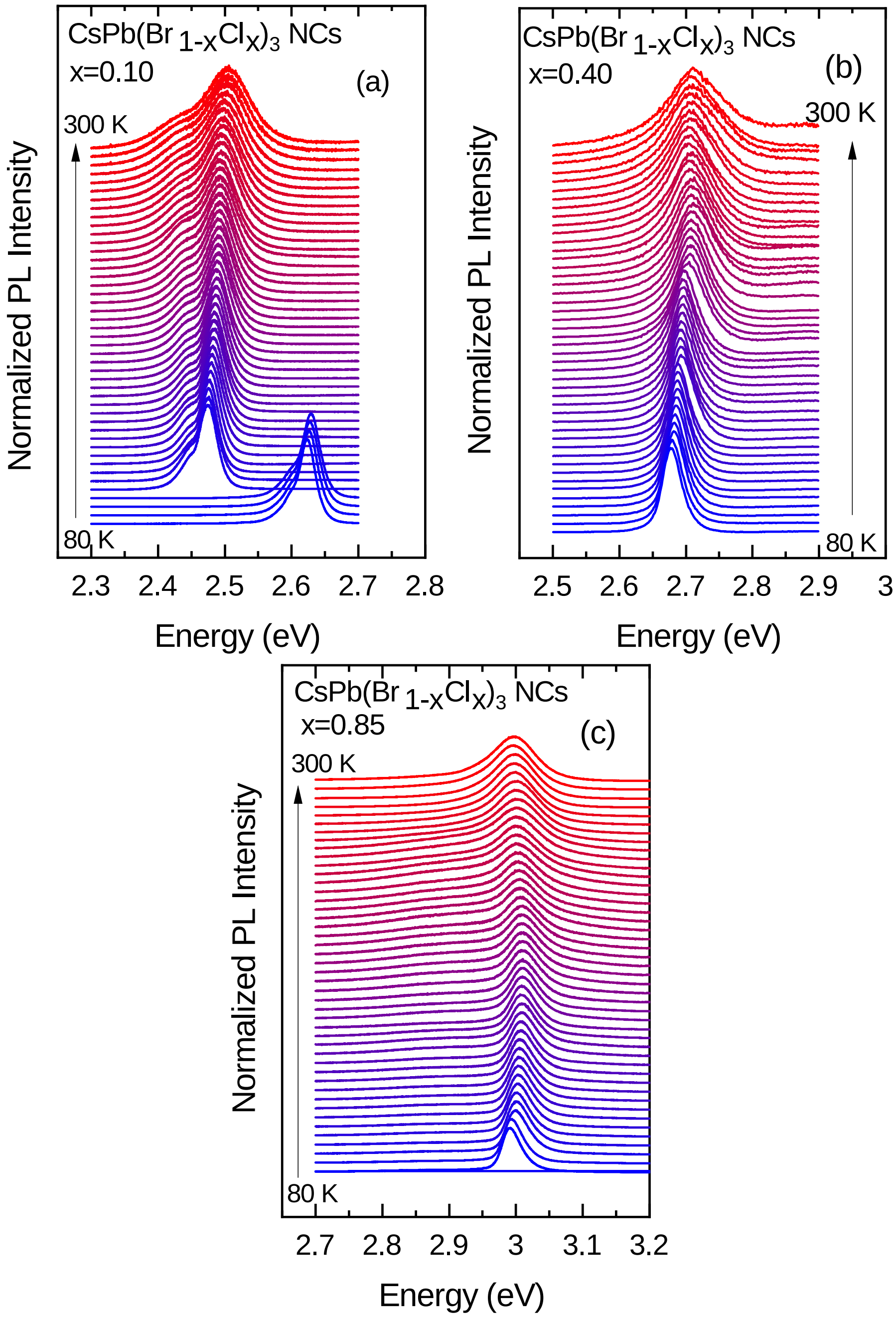

Figure S 5: PL spectra of $CsPb(Br_{1-x}Cl_x)_3$ mixed-anion NCs with (a) x=0.10, (b) x=0.40, and (c) x=0.85 measured at different temperatures between 80 and 300 K in steps of 5 K, using either the violet (405 nm) or ultraviolet (355 nm) laser line for excitation. The spectra were normalized to their maximum intensity and shifted vertically for clarity. We that the shoulder in the PL spectra for x=0.1 may arise from inhomogeneities (within the laser spot) either in NC size or crystalline phase. Indeed, from its temperature dependence (slight increase of peak energy with decreasing temperature), one can speculate that the shoulder could be due to the spurious presence of orthorhombic NCs.

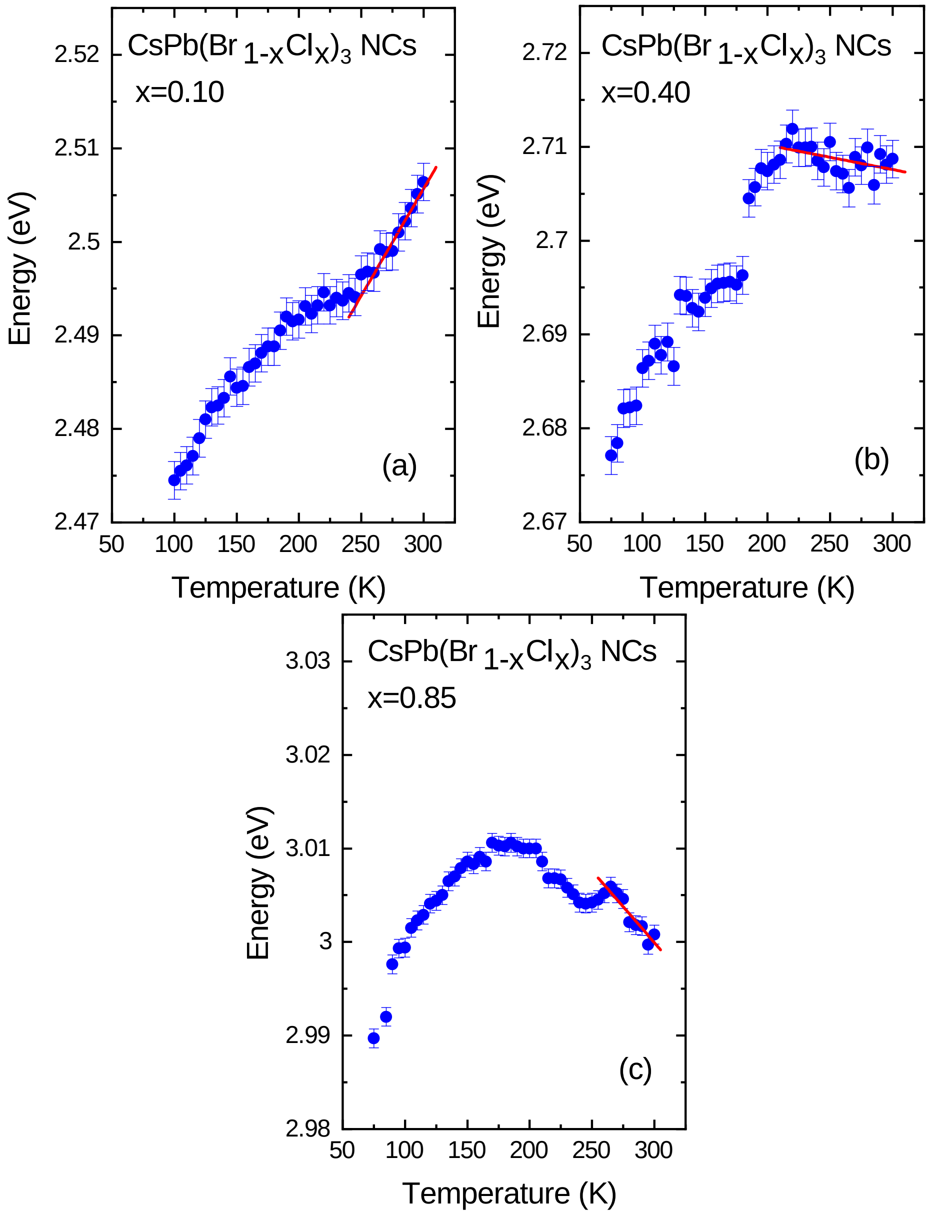

Figure S 6: PL peak energy position as a function of temperature (blue symbols) of $CsPb(Br_{1-x}Cl_x)_3$ mixed-anion NCs with (a) x=0.10, (b) x=0.40, and (c) x=0.85 in the temperature range between 80 and 300 K. The red lines represent the result of a linear fit to the data points around room temperature.

For low Cl contents below ca. 40% the main PL peak exhibits a gradual redshift and sharpening

with decreasing temperature (see also Figs. 2a and 2b of the main manuscript). Strikingly, the samples with high Cl content (above ca. 40%) exhibit initially a contrasting behavior: the PL peak first shifts to the blue until ca. 200 K, below which a red shift sets in with decreasing temperature. This clearly indicates that there is a sign turnover in the slope of the linear temperature dependence of the gap at a Cl concentration of about 40%.

Note ♯3: On the *linearity* of the gap temperature (T) and pressure (P) dependence in lead halide perovskites

In Ref.[2] we show that if a survey among lead halide perovskites is circumscribed to near ambient conditions, for which most of the materials crystallize either in a cubic or tetragonal phase, the observed T and/or P dependence of the gap is a *linear* function. In these phases, octahedral tilting does not play any role and the temperature and pressure behavior of the perovskite gap is solely determined by the effects of bond stretching. The latter strictly depend on the bonding/antibond and atomic orbital character of the electronic states at the top of the valence band and the bottom of the conduction band, but nothing else.[2,21,22] The typical trend is a gap that opens with increasing temperature but closes with increasing pressure. In the linearity range both temperature and pressure coefficients are constant, i.e. they are temperature and pressure independent.

Octahedral tilting, however, causes always an increase of the electronic gap, as a result of the effects of bond bending. The hybridization of the Pb 6*p* orbitals with the *s* orbitals of the corresponding halide anions imposes a serious constraint over the halide-Pb-halide bond to be straight. Any temperature or pressure-induced tilting of the corner-sharing PbX_6 octahedrons gradually reduces the X-Pb-X bond angle below 180° with the concomitant blue shift of the gap energy.[23–27] When bond-bending effects (octahedral tilting) compete with those due to bond stretching, this

leads to an outspoken non-linear temperature or pressure dependence of the gap. Please notice that in the case of the tetragonal and certain orthorhombic phases, although there is certain amount of (static) octahedral tilting already present in the crystal structure of the perovskite, this tilting does not change upon variation of temperature or application of pressure. Consequently, bond bending cannot be expressed, which leads to a linear variation of the gap as well. This is, for instance, the case of the orthorhombic phase of the $CsPb(Br_{1-x}Cl_x)_3$ NCs with Cl concentrations higher than ca. 40% stable at near-ambient conditions.

Note ♯4: The thermal expansion (TE) term

The effect on the gap due to the contraction of the lattice with decreasing temperature is intimately related to the response of the electronic band structure upon application of external hydrostatic pressure. It thus holds[18,19,28]

$$\left[\frac{\partial E_g}{\partial T}\right]_{TE} = -\alpha_V \cdot B_0 \cdot \frac{dE_g}{dP}, \tag{2}$$

where $-\alpha_V$ is the volumetric expansion coefficient, B_0 is the bulk modulus, i.e. the inverse of the compressibility, and $\frac{dE_g}{dP}$ is the pressure coefficient of the gap, which can be determined from high pressure experiments. At room temperature α_V is positive and the sign of Eq. (2) for the thermal expansion contribution is determined by the sign of the pressure coefficient. For most conventional semiconductor direct gaps $\frac{dE_g}{dP}$ is positive as well. Hence, thermal expansion causes a gap reduction. The lead halide perovskites are an exception, because the variation with pressure of the energy position of the PL peak measured with the DAC yields unusually large but negative pressure coefficient.[2] The negative sign of $\frac{dE_g}{dP}$ arises from the inverted atomic orbital character of the states at the top and bottom of the valence and conduction band of lead halide perovskites, respectively, as explained elsewhere.[22]

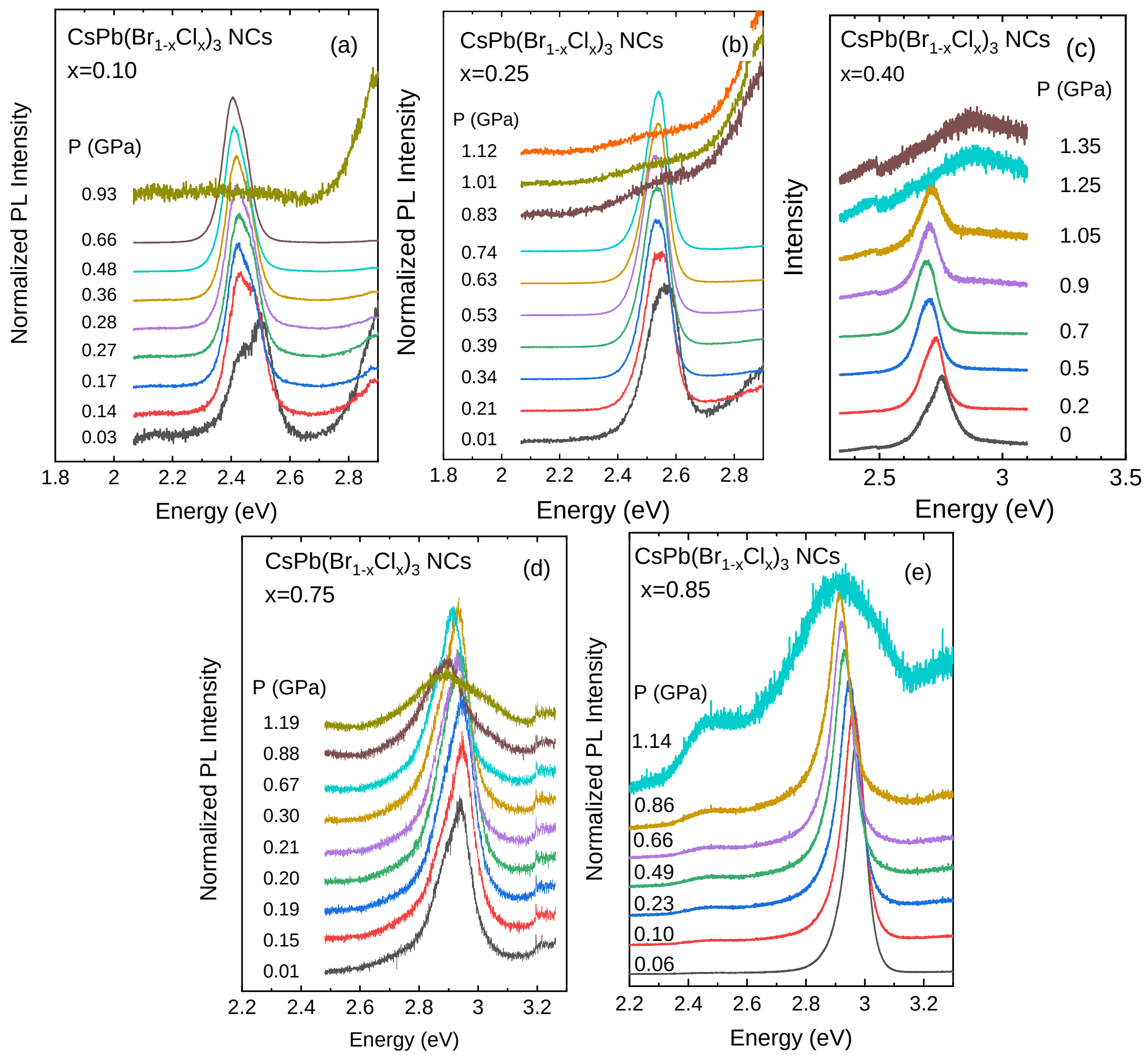

Figure S 7: PL spectra of $CsPb(Br_{1-x}Cl_x)_3$ mixed-anion NCs with (a) x=0.10, (b) x=0.25, (c) x=0.40, (d) x=0.75, and (e) x=0.85 measured at different pressures up to ca. 1.2 GPa, where the first phase transition occurs. Either the violet (405 nm) or ultraviolet (355 nm) laser line were used for excitation. The spectra were normalized to their maximum intensity and shifted vertically for clarity.

The gap pressure coefficient of Eq. (2) is determined from the slope of the energy versus pressure curves, where the data points were previously obtained from fits to the PL spectra recorded at different pressures. The cascades of PL spectra for different pressures in the range up to ca. 1

GPa, where the first phase transition occurs, are shown for the five compositions x=0.10, x=0.25, x=0.40, x=0.75, and x=0.85 in Figs. S7(a)-(e). In all cases the PL peak shifts initially to the red, thus, the pressure coefficient is negative.

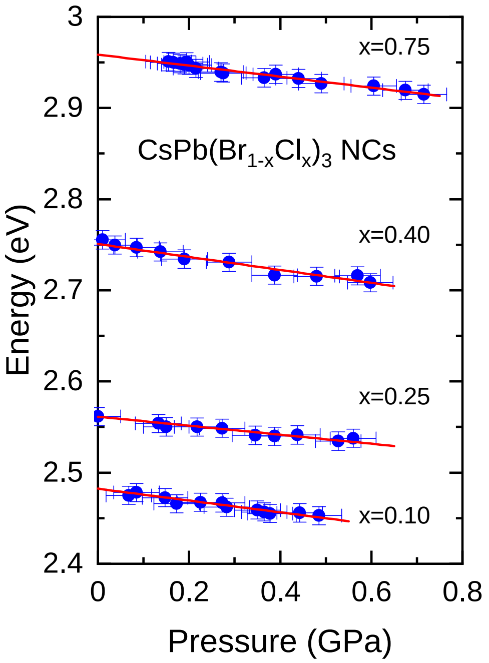

Figure S 8: The energy of the PL peak maximum (blue symbols) plotted as a function of pressure in the stability range of the ambient pressure phase of $CsPb(Br_{1-x}Cl_x)_3$ NCs for four representative compositions, as indicated. The red lines represent a fit to the data points using a linear function.

Figure S8 shows the variation with pressure of the gap energy, as obtained from PL line-shape fits, in the stability range of the ambient pressure phase. The slopes of the linear fits to the data points (red solid lines in Fig. S8) are listed in Table 1 of the manuscript for the different Cl concentrations studied here. Surprisingly, for all NC compositions the gap pressure coefficient $\frac{dE_g}{dP}$ is approx. the same in sign and magnitude, with an average value of (-60±15) meV/GPa (for a complete survey of the gap pressure and temperature coefficients of lead halide perovskites see Ref.[2]). Hence, with increasing temperature thermal expansion always causes a gradual opening of the gap, independent of chlorine content.

Note ♯5: The electron-phonon (EP) coupling term

Gopalan *et al.*[19] derived an expression for the energy shift of the gap induced by temperature, through electron-phonon interaction, of any electronic state $E_{n\mathbf{k}}$ with band index n and wavevector $\mathbf{k}$, for which all phonon modes of the branch j, wavevector $\mathbf{q}$ and frequency $\omega_{j\mathbf{q}}$ contribute:

$$\Delta E_{n\mathbf{k}}(T) = \sum_{j\mathbf{q}} \frac{\partial E_{n\mathbf{k}}}{\partial n_{j\mathbf{q}}} \left(n_{j\mathbf{q}}(T) + \frac{1}{2} \right), \tag{3}$$

where $n_{j\mathbf{q}} = \left(e^{\beta \hbar \omega_{j\mathbf{q}}} - 1 \right)^{-1}$ is the Bose-Einstein phonon occupation factor with $\beta = \frac{1}{k_B T}$. Importantly, the complex interaction coefficients $\frac{\partial E_{n\mathbf{k}}}{\partial n_{j\mathbf{q}}}$ contribute *both* to the energy shift of the bands as well as to a lifetime broadening of the electronic states (linewidths). Obviously, the sign of the renormalization of a gap is thus determined by the difference in magnitude and sign of the respective energy shift of valence and conduction band.

By invoking energy conservation, the summation in Eq. (3) transforms into an integral over the phonon frequencies:[19]

$$\begin{aligned} \Delta E_{n\mathbf{k}}(T) &= \int_0^{\infty} d\omega \cdot g^2F(n,\mathbf{k},\omega) \cdot \left(n_{j\mathbf{q}}(T) + \frac{1}{2} \right) \\ g^2F(n,\mathbf{k},\omega) &= \sum_{j\mathbf{q}} \frac{\partial E_{n\mathbf{k}}}{\partial n_{j\mathbf{q}}} \delta(\omega - \omega_{j\mathbf{q}}). \end{aligned} \tag{4}$$

The function $g^2F(n,\mathbf{k},\omega)$ is the so-called electron-phonon spectral function and is essentially the phonon density of states (DOS) appropriately weighted by electron-phonon matrix elements. As such, the spectral function is temperature independent, which means that the temperature dependence of the electron-phonon contribution to the gap shift arises solely from the Bose-Einstein occupation factor $n_{j\mathbf{q}}(T)$.

Equations. (3) and (4) indicate that the main contributions to the electron-phonon renormaliza-

tion of the gap arise from peaks in the DOS. In fact, this is at the origin of the Einstein-oscillator model introduced by Cardona and coworkers,[20,29,30] which approximate the $\frac{\partial E_{n\mathbf{k}}}{\partial n_{j\mathbf{q}}}$ coefficients by effective electron-phonon interaction parameters A_i for phonons with average frequency ω_i, inferred from the peaks in the phonon DOS. The EP correction to the gap then reads

$$[\Delta E_g(T)]_{EP} = \sum_i A_i \cdot \left(n_B(\omega_i, T) + \frac{1}{2} \right), \tag{5}$$

where n_B again stands for the Bose-Einstein occupation factor of the effective mode with frequency ω_i. The analytical expression for the EP term is simply obtained by taking the first derivative of the Bose-Einstein occupation number over temperature as:

$$\begin{aligned} \left[\frac{\partial E_g}{\partial T}\right]_{EP} &= \sum_i A_i \cdot \frac{\partial \left(n_B(\omega_i, T) + \frac{1}{2} \right)}{\partial T} \\ &= \sum_i \frac{A_i}{4T} \cdot \frac{\hbar \omega_i}{k_B T} \cdot \frac{1}{sinh^2 \left(\frac{\hbar \omega_i}{2 k_B T} \right)}. \end{aligned} \tag{6}$$

Here $n_B(\omega_i, T) = \left(e^{\beta \hbar \omega_i} - 1 \right)^{-1}$ with $\beta = \frac{1}{k_B T}$ stands for the Bose-Einstein phonon occupation factor of the ith oscillator.

To obtain the EP term, it is convenient to perform least-squares fits using the expression derived for the first derivative of the curves gap-energy-vs-temperature. In order to avoid the amplification of the scatter of the data points when taking the first derivative numerically and point-by-point, we have previously smoothed the E-vs-T curves, as shown for the five compositions studied in Figs. S9a-e. The resulting first-derivative data points (closed/open dark-green symbols) are shown in Figs. S10a-c for the samples with Cl content of x=0.1, 0.4 and 0.85, respectively.

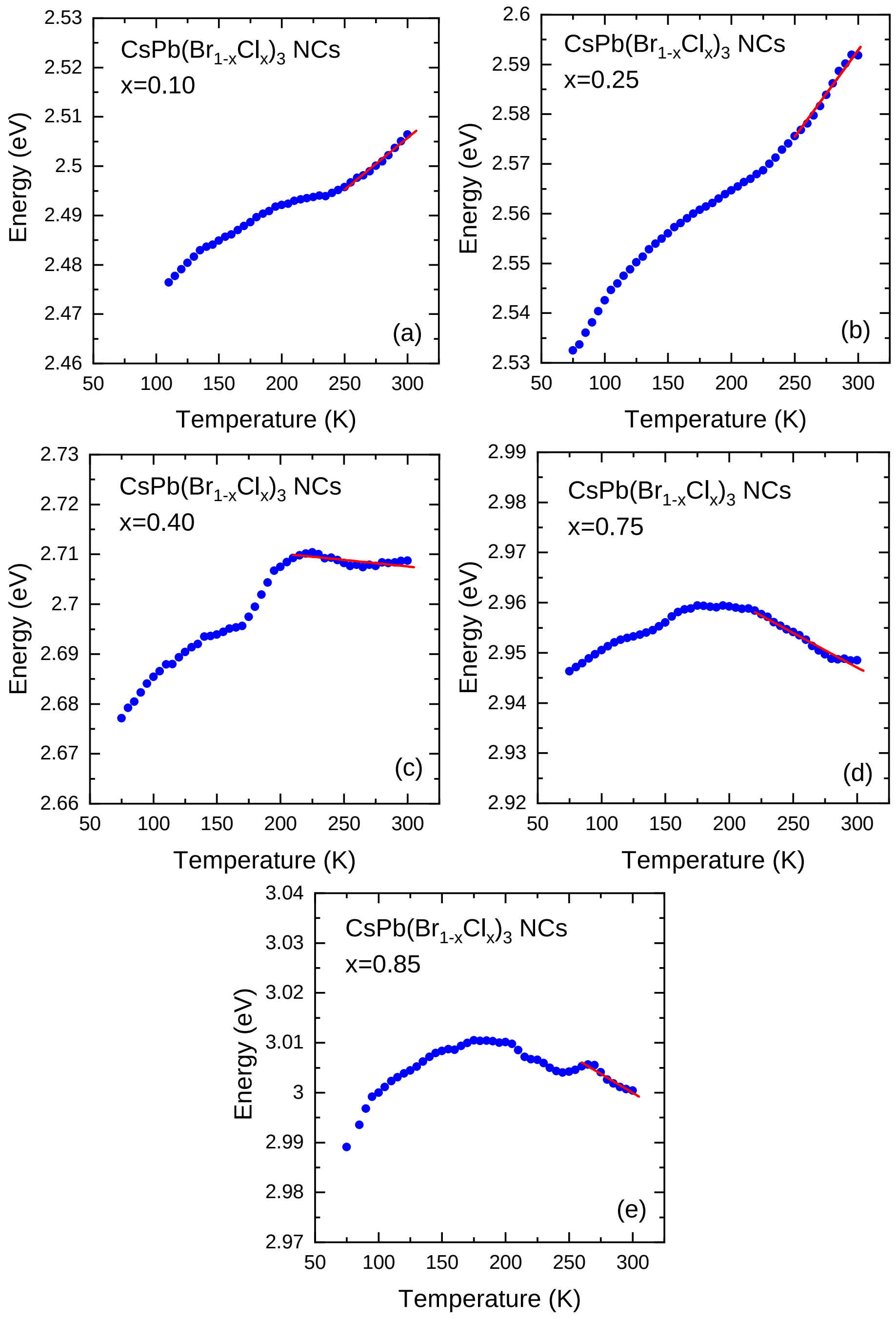

Figure S 9: PL peak energy position as a function of temperature (blue symbols), after smoothing, of $CsPb(Br_{1-x}Cl_x)_3$ mixed-anion NCs with (a) x=0.10, (b) x=0.25, (c) x=0.40, (d) x=0.75, and (e) x=0.85 in the temperature range between 80 and 300 K. The red lines represent the result of a linear fit to the data points around room temperature.

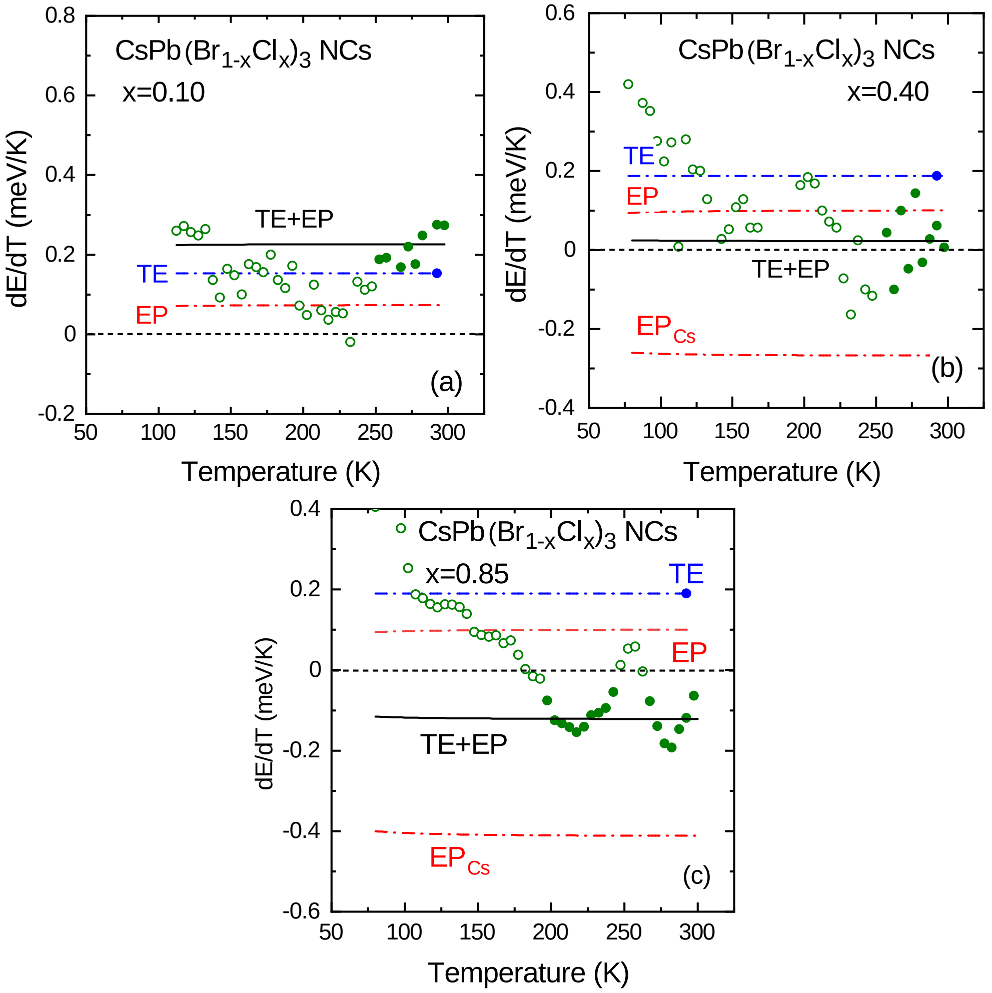

Figure S 10: The first derivative of the PL peak energy with respect to temperature (closed & open dark-green symbols), numerically calculated from the smoothed data of Fig. S9 for $CsPb(Br_{1-x}Cl_x)_3$ mixed-anion NCs with (a) x=0.10, (b) x=0.40, and (c) x=0.85. The solid black lines represent a fit only to the closed data points (linearity range), corresponding to the sum of the contribution of thermal expansion (TE, blue dot-dashed curve and symbol) and electron-phonon interaction (EP, red dot-dashed curve). In (b) and (c), the curve labeled EP_{Cs} represents the additional Einstein oscillator introduced to account for the anomalous electron-phonon coupling.

Table S 2: Einstein-oscillator parameters corresponding to the effective amplitude A_i and frequency ω_i describing the contribution from electron-phonon interaction $\left[\frac{\partial E_g}{\partial T}\right]_{EP}$ to the renormalization of the band gap in $CsPb(Br_{1-x}Cl_x)_3$ NCs. Numbers in parentheses are error bars (uncertainty of the last digits). Values marked with an asterisk are from $CsPbBr_3$ NCs.[2]

x	Einstein osc.	A_i (meV)	$\hbar\omega_i$ (meV)
0*	Osc. ♯1	6.7(5)*	6*
0.10	Osc. ♯1	4.5(5)	6
0.25	Osc. ♯1	12.5(5)	6
0.40	Osc. ♯1	7	6
	Osc. ♯2	-13(2)	4
0.75	Osc. ♯1	7	6
	Osc. ♯2	-20(1)	4
0.85	Osc. ♯1	7	6
	Osc. ♯2	-20(1)	4

Here, we would like to point out that the model with two Einstein oscillators has been previously used by Saran et al.[3] in an attempt to describe the temperature dependence of the gap of $CsPbI_3$ and $CsPbBr_3$ NCs in the temperature range from 5 to 300 K. The two oscillators were supposed to account for the coupling of electrons with acoustic and optical phonon modes of the perovskite, the latter mediated by Fröhlich interaction. Unfortunately, in this work two crucial issues were not taken into account. The first and most fundamental is that thermal expansion effects were ignored, probably misled by the results on cuprous halides[20] and silver chalcopyrites.[30] As previously pointed out by Francisco-López et al.,[31] discarding the TE term leads to an overestimation of the electron-phonon interaction, such that the outcome of its modeling in terms of Einstein

oscillators yielded unphysically large values for the frequencies of the involved phonons (larger than the cutoff frequency for the inorganic cage phonons which is roughly 25 meV). The second problem arises when the authors try to cover the temperature dependence of the gap over the entire range of the experiment for which the temperature dependence of the gap is outspoken non-linear. As deduced from the discussion in Note ♯3, such non-linearity arises to a large extent because the TE term is temperature dependent as well. Evidence of that are the different values obtained for the gap pressure coefficient of $MAPbI_3$ for different temperatures within the range of stability of the orthorhombic phase.[32] This is the reason for the need of two oscillators, i.e. to describe the nonlinearity, while we clearly show that, in the linearity regime around room temperature (constant TE term), the gap temperature renormalization of $CsPbBr_3$ NCs is perfectly described by a single oscillator for the EP term. Moreover, the electron-phonon coupling is in this case the *normal* one, as exhibited by most lead halide perovskites.[2]

Note ♯6: Normal electron-phonon coupling

As mentioned before, the normal EP coupling term is described in terms of a single Einstein-oscillator model, which contains two parameters: The oscillator amplitude A_{eff} and its characteristic frequency ω_{eff}. In principle, the latter should correspond to a given peak in the phonon density of states (DOS). For that purpose we reproduce the calculations of the phonon dispersion curves and the corresponding DOS from Ref.[14] The vibrational frequencies for the cubic phase of $MAPbBr_3$ were calculated within the harmonic phonon approximation using second order force constants obtained from density functional theory (DFT). Figure S11 (left panel) shows the phonon dispersions of the 18 inorganic cage modes within the harmonic approximation using a pseudo cubic lattice for $MAPbBr_3$ at room temperature. Negative-frequency soft modes are located around the Brillouin-zone boundary at the M and R points. The phonon dispersions are plotted considering

band crossings. The color refers to the nature of each phonon eigenmode. The three orthogonal acoustic modes are plotted in blue shades. The remaining modes are optical, plotted in groups of three with a similar shade for each orthogonal mode (these modes would be degenerate if the MA ions were replaced by a spherical atom). Figure S11 (right panel) displays the corresponding phonon DOS decomposed by sets of three orthogonal phonon eigenmodes, integrated over the full Brillouin zone, but not considering band crossings.

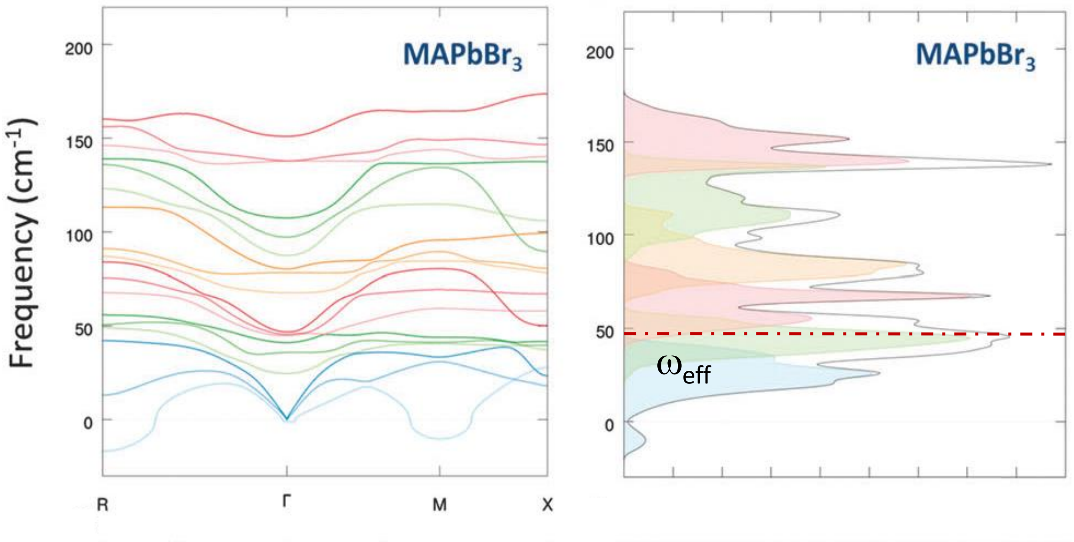

Figure S 11: (Left panel) Phonon dispersion curves and (Right panel) phonon density of states (DOS) of bulk $MAPbBr_3$ calculated within the harmonic phonon approximation using second order force constants obtained from density functional theory (adapted from Fig. 3 of Ref.[14]).

The dot-dashed horizontal line in the right panel of Fig. S11 represents the effective frequency of the Einstein oscillator used to describe the *normal* EP coupling term in the case of the $CsPbBr_3$ NCs. An inspection of the phonon DOS indicates that the oscillator frequency of 6 meV (i.e. ca. 48 cm^{-1}) lies slightly above the first well-defined peak or band in the DOS, corresponding to low-frequency optical modes partially intermixed with acoustical branches. Since these are all phonons of the inorganic cage, we can safely assume that a similar oscillator will account for the EP term

in the $CsPbBr_3$ NCs as well. This is so because both $MAPbBr_3$ and $CsPbBr_3$ share the same inorganic cage, being the influence of the A-site cation negligible to first order of approximation. Furthermore, we note that the contributions of the higher-frequency peaks in the phonon DOS are exponentially damped by the Bose-Einstein phonon occupation number. This explains why a single Einstein oscillator is enough to account for the coupling between the electrons and inorganic cage phonons.

Note ♯7: Anomalous electron-phonon coupling

Equation (4) in Note ♯5 tells us two important things about the electron-phonon interaction: it is an additive magnitude, given by an integral over frequency of the electron-phonon spectral function $g^2F(n,\mathbf{k},\omega)$, and that there has to be a non-vanishing overlap between the electronic and vibrational wavefunctions for the matrix elements $\frac{\partial E_{n\mathbf{k}}}{\partial n_{j\mathbf{q}}}$ to be different from zero. Since the states near the valence and conduction band edges in lead halide perovskites are completely confined to the inorganic cage, the EP term only captures contributions from lattice phonons, excluding the A-site cations. This is at the origin of the *normal* EP coupling term, represented by the Einstein oscillator with positive amplitude. However, the A-site cations might also add oscillator strength into the electron-phonon spectral function by coupling indirectly with the charge carriers in the inorganic cage. A way in which this indirect coupling can proceed is when a translational, rotational or librational degree of freedom of the A-site cations enters into resonance with any vibrational mode of the inorganic cage lattice. In the case of Cs being the A-site cation, the modes participating in the anomalous (indirect) EP coupling are the Cs rattlers,[33] as explained in the main manuscript. Due to the Coulombic nature of the anion-cation attraction, the most likely phonon modes involved in the indirect coupling are the ones associated to octahedral tilting. Please notice that this implies the formation of modes featuring *coherence* between the A-site cation motion (Cs rattlers, in this

case) and the octahedral-tilting phonons. This leads at last to the *anomalous* EP term which is accounted for within our model by the second Einstein oscillator with negative amplitude. We point out that incoherent coupling can only affect the lifetime (even the frequency) of the inorganic cage phonons[15] but never add oscillator strength to the EP spectral function.

Further support for the idea that octahedral tilting is involved in the anomalous EP coupling comes from recent first-principles density functional theory calculations of the energetics of octahedral tilting in $CsPbI_3$ and $CsSnI_3$.[34] The constant-energy surfaces of the atomic potential energy, which corresponds to the sum of the electronic kinetic energy and the potential energies due to electron-electron, electron-nucleus and nucleus-nucleus interactions,[35] were calculated as a function of octahedral tilting angle for both the case of in-phase and out-of-phase tilting between adjacent octahedrons.[34] In the orthorhombic *Pnma* phase, these potential energy surfaces exhibit multi-well landscape with four well-defined local minima separated by relatively small activation barriers, when tilting is only considered in a plane. At high enough temperatures, the atomic wave function delocalizes between the equivalent minima, leading to a dynamic tilt situation, which is more or less fast depending on the height of the barriers.[34,35] In contrast, at low temperatures the atoms localize at one of the four equivalent minima, which corresponds to a situation of static (fixed) octahedral tilting. In the work of Pérez-Fidalgo et al.,[36] it was proposed that the anomalous EP term needed to describe the reduction in temperature slope of the gap of low-content $Cs_xMA_{1-x}PbI_3$, occurring at around ambient temperature, arises from dynamic tilting fluctuations of the PbI_6^- octahedrons in synchrony with the motion of the Cs^+ cations. This was observed at temperatures higher than the onset temperature for unfolding the Cs^+ cation dynamics.

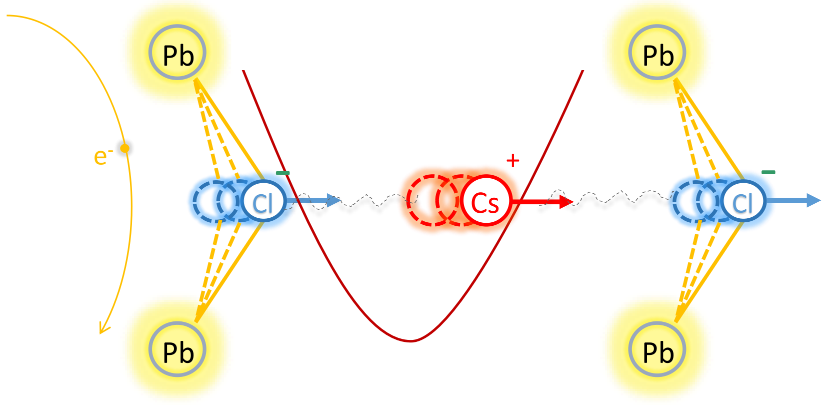

Figure S 12: Sketch illustrating the anomalous EP term arising from the (in-phase) coherent coupling of phonon modes of the inorganic cage involving octahedral tilting and the oscillations of the Cs cations in the potential energy well within the cage voids.

In contrast, a different situation is observed for $CsPb(Br,Cl)_3$ NCs, because the anomalous EP coupling appears for the shrunken orthorhombic phase at high Cl concentrations, for which the octahedral tilting is static and the dynamics of the Cs^+ cations is frozen. Nevertheless, we rationalize this finding as due to the reduced steric volume left for the Cs^+ cations inside the cage voids of the orthorhombic phase. As illustrated in the sketch of Fig. S12, coherence between the vibrations involving tilting and Cs oscillations in the potential well of the void seems to be established via the attractive anion-cation interaction. On the contrary, for the cubic Br-rich NCs the constant energy surfaces exhibit a single potential well centered at zero tilt, as is mandatory for a cubic phase. Consequently, although the dynamics of the Cs cation is unfolded, the motion is restricted to the central region of the cage voids and is characterized by a spherical atomic probability density cloud. This, combined with the larger cage size, could be the cause of the loss of coherence, so only the normal EP coupling remains; the one involving only the phonons of the inorganic cage.

In order to visualize the rattler modes involved in the anomalous, indirect EP coupling, we reproduce in Fig. S13 the results for the harmonic phonon dispersion relations of cubic $CsPbBr_3$ from large-scale molecular dynamics (MD) simulations combined with machine-learning interatomic potentials.[33] By highlighting in red color the Cs contribution in the respective eigenmode, one can identify nearly dispersionless rattling bands. In the middle panel, one can see that the Cs cations mainly contribute to the phonon band structure in an energy window around ca. 1 THz ($\approx$ 4 mev). This frequency perfectly matches that of acoustic phonons of the inorganic cage, which exhibit *in-phase* octahedral tilting.[14] In the left(right) panel, the masses of only the Cs atoms were artificially lowered(raised), which results in a blue(red)-shift of the central rattling frequency, respectively. For the light Cs cations, the central frequency shifts up to about 2.2 THz, thereby almost removing the overlap with the linear acoustic bands.

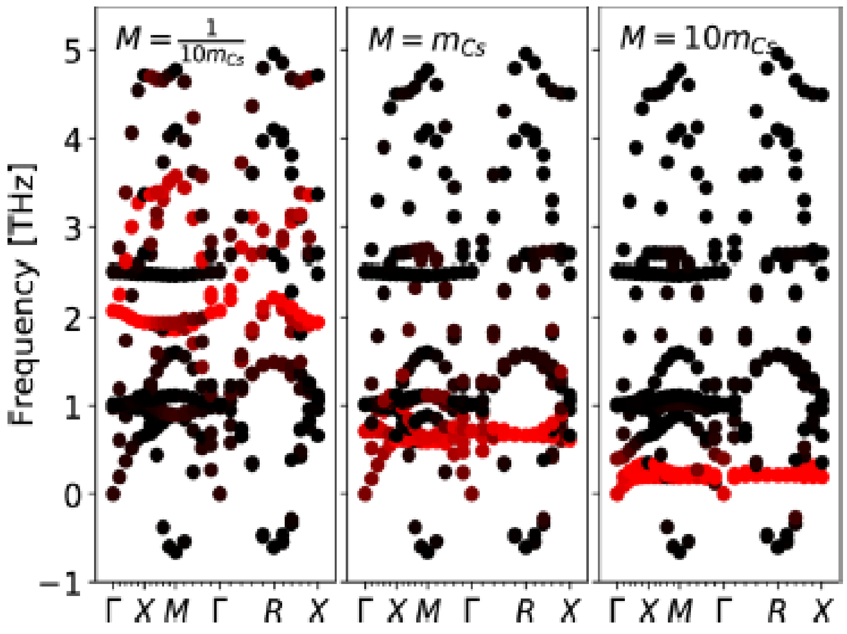

Figure S 13: Shifting the rattling band. Harmonic phonon band structure of cubic $CsPbBr_3$ with light ($m_{Cs}/10$), normal (m_{Cs}), and heavy ($m_{Cs} \cdot 10$) effective mass of the Cs cation. The color scale from black to red indicates the increasing Cs contribution to the respective eigenmode (adapted from Fig. 2 of Ref.[33]).

Finally, we discuss the issue of the negative sign of the anomalous EP term. A careful analysis of the exciton line broadening and the phonon-related features in the low-temperature PL spectra of $FAPbI_3$ NCs, where FA stands for formamidinium,[37] revealed that the leading electron-phonon interaction is of the Fröhlich type.[38] This is not surprising given the polar character of the vibrations in metal halide perovskites. The Fröhlich interaction accounts for the Coulombic coupling between charge carriers and the vibrating anions/cations. The sign reversal thus simply comes from the fact that if the electron-anion interaction is repulsive, then the electron-(A-site)cation interaction is attractive, and viceversa. The latter is at work by the Cs rattler modes leading to the anomalous EP coupling.

References

(1) Tong, Y.; Bladt, E.; Aygüler, M. F.; Manzi, A.; Milowska, K. Z.; Hintermayr, V. A.; Docampo, P.; Bals, S.; Urban, A. S.; Polavarapu, L.; Feldmann, J., Highly Luminescent Cesium Lead Halide Perovskite Nanocrystals with Tunable Composition and Thickness by Ultrasonication. *Angew. Chem. Int. Ed.* **2016**, 55, 13887-13892.

(2) Fasahat, S.; Schäfer, B.; Xu, K.; Fiuza-Maneiro, N.; Gómez-Graña, S.; Alonso, M. I.; Polavarapu, L.; Goñi, A. R. Absence of Anomalous Electron-Phonon Coupling in the Temperature Renormalization of the Gap of $CsPbBr_3$ Nanocrystals. *J. Phys. Chem. C* **2025**, 129, 453-463.

(3) Saran, R.; Heuer-Jungemann, A.; Kanaras, A. G.; Curry, R. J. Giant Bandgap Renormalization and Exciton-Phonon Scattering in Perovskite Nanocrystals. *Adv. Optical Mater.* **2017**, 5, 1700231/1-9.

(4) Fan, T.; Lü, J.; Chen, Y.; Yuan, W.; Huang, Y., Random lasing in Cesium Lead Bromine Perovskite Quantum Dots Film. *J. Mater. Sci.: Mater. Electron.* **2018**, 30, 1084-1088.

(5) Chen, L.-C.; Kao, C.-H., Improved Extraction Efficiency of $CsPbBr_3$ Perovskite Light-Emitting Diodes due to Anodic Aluminum Oxide Nanopore Structure. *Sci. Rep.* **2022**, 12, 14750.

(6) Wang, Y.; Yang, F.; Li, X.; Ru, F.; Liu, P.; Wang, L.; Ji, W.; Meng, X.-M.; Meng, X., Epitaxial Growth of Large-Scale Orthorhombic $CsPbBr_3$ Perovskite Thin Films with Anisotropic Photoresponse Property. *Adv. Funct. Mater.* **2019**, 29, 1904913.

(7) Toso, S.; Baranov, D.; Filippi, U.; Giannini, C.; Manna, L., Collective Diffraction Effects in Perovskite Nanocrystal Superlattices. *Acc. Chem. Res.* **2023**, 56, 66-76.

(8) Hoffman, A. E.; Saha, R. A.; Borgmans, S.; Puech, P.; Braeckevelt, T.; Roeffaers, M. B.; Steele, J. A.; Hofkens, J.; Van Speybroeck, V. Understanding the Phase Transition Mechanism in the Lead Halide Perovskite $CsPbBr_3$ via Theoretical and Experimental GIWAXS and Raman Spectroscopy. *APL Mater.* **2023**, 11, 041124.

(9) Brennan, M. C.; Kuno, M.; Rouvimov, S. Crystal structure of individual $CsPbBr_3$ perovskite nanocubes. *Inorg. Chem.* **2018**, 58, 1555-1560.

(10) López, C. A.; Abia, C.; Alvarez-Galván, M. C.; Hong, B.-K.; Martínez-Huerta, M. V.; Serrano-Sánchez, F.; Carrascoso, F.; Castellanos-Gómez, A. s.; Fernández-Díaz, M. T.; Alonso, J. A. Crystal Structure Features of $CsPbBr_3$ Perovskite Prepared by Mechanochemical Synthesis. *ACS Omega* **2020**, 5, 5931-5938.

(11) Cottingham, P.; Brutchey, R. L. On the Crystal Structure of Colloidally Prepared $CsPbBr_3$ Quantum Dots. *Chem. Commun.* **2016**, 52, 5246-5249.

(12) Boehme, S. C.; Bodnarchuk, M. I.; Burian, M.; Bertolotti, F.; Cherniukh, I.; Bernasconi, C.; Zhu, C.; Erni, R.; Amenitsch, H.; Naumenko, D. Strongly confined $CsPbBr_3$ quantum dots as quantum emitters and building blocks for rhombic superlattices. *ACS Nano* **2023**, 17, 2089-2100.

(13) Bertolotti, F.; Dengo, N.; Cervellino, A.; Bodnarchuk, M. I.; Bernasconi, C.; Cherniukh, I.; Berezovska, Y.; Boehme, S. C.; Kovalenko, M. V.; Masciocchi, N.; Guagliardi, A. Size- and Temperature-Dependent Lattice Anisotropy and Structural Distortion in $CsPbBr_3$ Quantum Dots by Reciprocal Space X-ray Total Scattering Analysis. *Small Struct.* **2024**, 5, 2300264/1-15.

(14) Leguy, A. M. A.; Goñi, A. R.; Frost, J. M.; Skelton, J.; Brivio, F.; Rodríguez-Martínez, X.;

Weber, O. J.; Pallipurath, A.; Alonso, M. I.; Campoy-Quiles, M.; Weller, M. T.; Nelson, J.; Walsh, A.; Barnes, P. R. F. Dynamic Disorder, Phonon Lifetimes, and the Assignment of Modes to the Vibrational Spectra of Methylammonium Lead Halide Perovskites. *Phys. Chem. Chem. Phys.* **2016**, 18, 27051-27066.

(15) Goñi, A. R. Raman Linewidths as a Probe of Lattice Anharmonicity and Dynamic Disorder in Metal Halide Perovskites. *Asian J. Phys.* **2024**, 33, 29-38.

(16) Yaffe, O.; Guo, Y.; Tan, L. Z.; Egger, D. A.; Hull, T.; Stoumpos, C. C.; Zheng, F.; Heinz, T. F.; Kronik, L.; Kanatzidis, M. G.; et al. Local Polar Fluctuations in Lead Halide Perovskite Crystals. *Phys. Rev. Lett.* **2017**, 118, 136001.

(17) Hansen, K. R.; Colton, J. S.; Whittaker-Brooks, L. Measuring the Exciton Binding Energy: Learning from a Decade of Measurements on Halide Perovskites and Transition Metal Dichalcogenides. *Adv. Optical Mater.* **2024**, 12, 2301659/1-136.

(18) Lautenschlager, P.; Allen, P. B.; Cardona, M. Temperature Dependence of Band Gaps in Si and Ge. *Phys. Rev. B* **1985**, 31, 2163-2171.

(19) Gopalan, S.; Lautenschlager, P.; Cardona, M. Temperature Dependence of the Shifts and Broadenings of the Critical Points in GaAs. *Phys. Rev. B* **1987**, 35, 5577-5584.

(20) Göbel, A.; Ruf, T.; Cardona, M.; Lin, C. T.; Wrzesinski, J.; Steube, M.; Reimann, K.; Merle, J.-C.; Joucla, M. Effects of the isotopic composition on the fundamental gap of CuCl. *Phys. Rev. B* **1998**, 57, 15183-15190.

(21) Goñi, A. R.; Syassen, K. Optical Properties of Semiconductors Under Pressure. *Semicond. Semimetals* **1998**, 54, 247-425, and references therein.

(22) Francisco López, A.; Charles, B.; Weber, O. J.; Alonso, M. I.; Garriga, M.; Campoy-Quiles, M.; Weller, M. T.; Goñi, A. R. Pressure-Induced Locking of Methylammonium Cations Versus Amorphization in Hybrid Lead Iodide Perovskites. *J. Phys. Chem. C* **2018**, 122, 22073-22082.

(23) Kong, L.; Liu, G.; Gong, J.; Hu, Q.; Schaller, R. D.; Dera, P.; Zhang, D.; Liu, Z.; Yang, W.; Tang, Y.; Wang, C.; Wei, S.-H.; Xu, T.; Mao, H.-K. Simultaneous Band-Gap Narrowing and Carrier-Lifetime Prolongation of Organic-Inorganic Trihalide Perovskites. *PNAS* **2016**, 113, 8910-8915.

(24) Jaffe, A.; Lin, Y.; Beavers, C. M.; Voss, J.; Mao, W. L.; Karunadasa, H. I. High-Pressure Single-Crystal Structures of 3D Lead-Halide Hybrid Perovskites and Pressure Effects on their Electronic and Optical Properties. *ACS Cent. Sci.* **2016**, 2, 201-209.

(25) Jaffe, A.; Lin, Y.; Karunadasa, H. I. Halide Perovskites under Pressure: Accessing New Properties Through Lattice Compresion. *ACS Energy Lett.* **2017**, 2, 1540-1555.

(26) Ghosh, D.; Walsh Atkins, P.; Islam, M. S.; Walker, A. B.; Eames, C. Good Vibrations: Locking of Octahedral Tilting in Mixed-Cation Iodide Perovskites for Solar Cells. *ACS Energy Lett.* **2017**, 2, 2424-2429.

(27) Ghosh, D.; Aziz, A.; Dawson, J. A.; Walker, A. B.; Islam, M. S. Putting the Squeeze on Lead Iodide Perovskites: Pressure-Induced Effects to Tune Their Structural and Optoelectronic Behavior. *Chem. Mater.* **2019**, 31, 4063-4071.

(28) Cardona, M. Electron-Phonon Interaction in Tetrahedral Semiconductors. *Solid State Commun.* **2005**, 133, 3-18.

(29) Serrano, J.; Schweitzer, Ch.; Lin, C. T.; Reimann, K.; Cardona, M.; Fröhlich, D. Electron-Phonon Renormalization of the Absorption Edge of the Cuprous Halides. *Phys. Rev. B* **2002**, 65, 125110/1-7.

(30) Bhosale, J.; Ramdas, A. K.; Burger, A.; Muñoz, A.; Romero, A. H.; Cardona, M.; Lauck, R.; Kremer, R. K. Temperature Dependence of Band Gaps in Semiconductors: Electron-Phonon Interaction. *Phys. Rev. B* **2012**, 86, 195208/1-10.

(31) Francisco López, A.; Charles, B.; Weber, O. J.; Alonso, M. I.; Garriga, M.; Campoy-Quiles, M.; Weller, M. T.; Goñi, A. R. Equal Footing of Thermal Expansion and Electron-Phonon Interaction in the Temperature Dependence of Lead Halide Perovskite Band Gaps. *J. Phys. Chem. Lett.* **2019**, 10, 2971-2977.

(32) Pieniazek, A.; Dybala, F.; Polak, M. P.; Przypis, L.; Herman, A. P.; Kopaczek, J.; Kudrawiec, R. Bandgap Pressure Coefficient of a $CH_3NH_3PbI_3$ Thin Film Perovskite. *J. Phys. Chem. Lett.* **2023**, 14, 6470-6476.

(33) Lahnsteiner, J.; Rang, M.; Bokdam, M. Tuning Einstein Oscillator Frequencies of Cation Rattlers: A Molecular Dynamics Study of the Lattice Thermal Conductivity of $CsPbBr_3$. *J. Phys. Chem. C* **2024**, 128, 1341-1349.

(34) Klarbring, J. Low-Energy Paths for Octahedral Tilting in Inorganic Halide Perovskites. *Phys. Rev. B* **2019**, 99, 104105/1-7.

(35) Adams, D. J.; Churakov, S. V. Classification of Perovskite Structural Types with Dynamical Octahedral Tilting. *Int. Union Crystall. J.* **2023**, 10, 1-12.

(36) Pérez-Fidalgo, L.; Xu, K.; Charles, B. L.; Henry, P. F.; Weller, M. T.; Alonso, M. I.; Goñi,

A. R. Anomalous Electron-Phonon Coupling in Cesium-Substituted Methylammonium Lead Iodide Perovskites. *J. Phys. Chem. C* **2023**, 127, 22817-22826.

(37) Fu, M.; Tamarat, P.; Trebbia, J.-B.; Bodnarchuk, M. I.; Kovalenko, M. V.; Even, J.; Lounis, B. Unraveling Exciton-Phonon Coupling in Individual $FAPbI_3$ Nanocrystals Emitting Near-Infrared Single Photons. *Nat. Commun.* **2018**, 9, 3318/1-10.

(38) Yu, P. Y.; Cardona, M. *Fundamentals of Semiconductors*, 4th Edt. (Springer, Heidelberg, 2010).
