# Supplementary material for: Sign of the Gap Temperature Dependence in CsPb(Br,Cl)3 Nanocrystals Determined by Cs-Rattler-Mediated Electron–Phonon Coupling
Source: J Phys Chem Lett. 2025 Jan 23;16(4):1134–41. doi: 10.1021/acs.jpclett.4c03491 (PMC11915369; doi:10.1021/acs.jpclett.4c03491)
Supplement: Supplementary file 2 — jz4c03491_si_002.pdf [file jz4c03491_si_002.pdf]

jz-2024-03491g.R1

Name: Peer Review Information for "Sign of the Gap Temperature Dependence in CsPb(Br,Cl)₃ Nanocrystals Determined by Cs-Rattler-Mediated Electron-Phonon Coupling"

First Round of Reviewer Comments

Reviewer: 1

Comments to the Author

Goñi and colleagues' work evaluates the inversion in the band gap temperature-dependence behaviour observed in different ABX3 perovskite nanocrystals with high and low contents of chloride. For the purpose of the study, they synthesized CsPbBr(1-x)Clx nanocrystals with different x compositions. The study is meticulously planned, with an exhaustive spectroscopic characterization of the samples followed by a solid-state analysis that is well-founded. The authors have a strong background in this matter, as evidenced by their recent publications, and the effort they have made to clarify their analysis in the supporting information is appreciated.

After reading of the manuscript, I have several questions:

1- DRX diffractions of high bromide content nanocrystals present narrow peaks. How does this correlate with the 10 nm crystal sizes revealed by TEM?

2- The absence of the strong diffraction at $2\theta = 21$ (e.g. PDF card ICSD 29073) in the nanocrystals obtained by the authors, despite its detection in both orthorhombic and cubic systems, raises questions regarding the presence of any preferential growth

3- Does the ligands used in this work affect the analysis carried on in the work?

4- The experimental details of the synthesis, including the sample preparation for the optical characterization should be included in the SI.

5- In the case of a composition of x=0.1, the PL demonstrates an additional peak situated at a lower energy level in comparison to the principal peak (see Fig. S5a). What could be the origin of this peak?

Author's Response to Peer Review Comments:

Response letter for Editor and Reviewers

Dear Editor

Senior Editor of the Journal of Physical Chemistry Letters

Thank you very much for your email with the referee report on our manuscript entitled *Sign of the Gap Temperature Dependence in CsPb(Br,Cl)*$_3$ *Nanocrystals Determined by Cs-Rattler-Mediated Electron-Phonon Coupling*. We would like to thank the reviewer for the very positive assessment of our work and the constructive comments, which have led to an improvement of the manuscript. We are particularly pleased that the reviewer considers that our work was meticulously planned, with an exhaustive spectroscopic characterization of the samples followed by a well-founded solid-state analysis, so he/she recommends publication in JPCL, albeit after revision.

We provide below a point-by-point answer to the reviewer's comments, together with a list of the changes introduced in the manuscript for its improvement. We look forward to the publication of the revised version of our paper in the Journal of Physical Chemistry Letters, as we believe we have adequately addressed the criticism raised by the reviewer and improved the presentation of the results accordingly.

Sincerely yours,

Alejandro Gon˜i (on behalf of all coauthors)

Response to the Reviewer:

1. Reviewer comment:

1. DRX diffractions of high bromide content nanocrystals present narrow peaks. How does thiscorrelate with the 10 nm crystal sizes revealed by TEM?

Authors reply:

First of all, we would like to thank the referee for the positive assessment of our work and the constructive comments. Regarding the narrow half widths (ca. 0.1 deg.) of the X-ray diffraction peaks in the 2θ scans of the NCs with high Br concentration, we are led to the conclusion that they are of instrumental origin. We have checked that for the X-ray wavelength used (approx. 0.15 nm) a broadening of the order of the peak half width would be caused only if the size of the NCs is smaller than 5 nm.

2. *Reviewer comment:*

2. The absence of the strong diffraction at 2θ = 21 (e.g. PDF card ICSD 29073) in the nanocrystals obtained by the authors, despite its detection in both orthorhombic and cubic systems, raises questions regarding the presence of any preferential growth.

Authors reply:

We thank the referee for pointing that out and acknowledge that this is a valid concern. Typically, this peak is reported in the literature as the one with the highest intensity. However, numerous studies have shown that the intensity of the peak corresponding to the (1 1 0) plane can diminish significantly,[1,2] and in some instances, become nearly imperceptible.[3] This phenomenon can be attributed to the formation of superlattices[4] resulting from the drop-casting of NCs onto the glass substrate. Consequently, the alignment of the crystals in a specific orientation may obscure certain information about their crystal faces.

1. Fan, T.; L, J.; Chen, Y.; Yuan, W.; Huang, Y., Random lasing in cesium lead bromine perovskitequantum dots film. Journal of Materials Science: Materials in Electronics 2019, 30, 1084-1088.
2. Chen, L.-C.; Kao, C.-H., Improved extraction efficiency of CsPbBr3 perovskite light-emitting diodesdue to anodic aluminum oxide nanopore structure. Scientific Reports 2022, 12, 14750.
3. Wang, Y.; Yang, F.; Li, X.; Ru, F.; Liu, P.; Wang, L.; Ji, W.; Meng, X.-M.; Meng, X., Epitaxial Growth of Large?Scale Orthorhombic CsPbBr 3 Perovskite Thin Films with Anisotropic Photoresponse Property. Advanced Functional Materials 2019, 29, 1904913.
4. Toso, S.; Baranov, D.; Filippi, U.; Giannini, C.; Manna, L., Collective Diffraction Effects in PerovskiteNanocrystal Superlattices. Accounts of chemical research 2023, 56, 66-76.

Changes in the Supporting Information:

By the discussion of the X-ray data, we added the following paragraph, including four new references: ”At this point, we would like to comment on the absence of strong diffraction at 2θ = 21 deg. (e.g. PDF card ICSD 29073). Typically, this peak is reported in the literature as the one with the highest intensity. However, numerous studies have shown that the intensity of the peak corresponding to the (110) plane can diminish significantly [4,5] and, in some instances, become nearly imperceptible [6]. This phenomenon can be attributed to the formation of superlattices [7] resulting from the drop-casting of the NCs onto the glass substrate. Consequently, the alignment of the crystals in a specific orientation may obscure certain information about their crystal faces.”

3. Reviewer comment:

3. Does the ligands used in this work affect the analysis carried on in the work?

Authors reply:

We have no evidence that the particular ligands used for the stabilization of our colloidal NCs play a role in their light-emitting properties.

4. *Reviewer comment:*

4. The experimental details of the synthesis, including the sample preparation for the optical characterization should be included in the SI.

Authors reply:

Thanks for pointing that out. We now added an entire section (**Note *1*0**) to the SI with details about the employed materials and the synthesis of the NCs, including the way in which the variation of the halide composition was performed by ionic exchange.

5. *Reviewer comment:*

5. In the case of a composition of x=0.1, the PL demonstrates an additional peak situated at a lowerenergy level in comparison to the principal peak (see Fig. S5a). What could be the origin of this peak?

Authors reply:

We believe that the shoulder observed in the PL spectra of the sample with Cl composition x=0.1 arises from inhomogeneities (within the laser spot) either in the NC size or in the crystalline phase.

Indeed, from its temperature dependence (slight increase of peak energy with decreasing temperature), one can speculate that the shoulder could be indicative of the spurious presence of NCs with orthorhombic crystal structure. We now comment on this issue in the SI.
